# Modifiable lifestyle factors in early life and inflammatory bowel disease risk in two birth cohorts

**DOI:** 10.1093/ecco-jcc/jjag114

**Published:** 2026-08-24

**Authors:** Annie Guo, Ida Sigvardsson, Henrik Imberg, Tereza Lerchova, Johnny Ludvigsson, Ketil Størdal, Karl Mårild

**Affiliations:** Department of Pediatrics, Institute of Clinical Sciences, Sahlgrenska Academy, University of Gothenburg, Gothenburg, Vitaminvägen 26, Gothenburg, 41650, Sweden; Department of Pediatrics, Institute of Clinical Sciences, Sahlgrenska Academy, University of Gothenburg, Gothenburg, Vitaminvägen 26, Gothenburg, 41650, Sweden; Statistiska Konsultgruppen Sweden AB, Gothenburg, Stigbergsliden 5, Gothenburg, 414 63, Sweden; Department of Molecular and Clinical Medicine, Institute of Medicine, Sahlgrenska Academy, University of Gothenburg, Gothenburg, Blå stråket 5 B Wallenberglab/SU, Gothenburg, 41345, Sweden; Department of Pediatrics, Institute of Clinical Sciences, Sahlgrenska Academy, University of Gothenburg, Gothenburg, Vitaminvägen 26, Gothenburg, 41650, Sweden; Division of Pediatrics, Biomedical and Clinical Sciences, Linköping University, Linköping, Campus US, Universitetssjukhuset, Linköping, 581 85, Sweden; Crown Princess Victoria Children’s Hospital, Region Östergötland, Linköping, H.K.H. Kronprinsessan Victorias barn- och ungdomssjukhus Universitetssjukhuset, Linköping, 581 85, Sweden; Pediatric Research Institute, Oslo University Hospital and Faculty of Medicine, University of Oslo, Oslo, Kirkeveien 166 Bygg 9 OUS Ullevål, Oslo, 0450, Norway; Department of Child and Adolescent Health, Oslo University Hospital, Oslo, Kirkeveien 166, Oslo, 0450, Norway; Department of Pediatrics, Institute of Clinical Sciences, Sahlgrenska Academy, University of Gothenburg, Gothenburg, Vitaminvägen 26, Gothenburg, 41650, Sweden; Department of Pediatrics, Queen Silvia Children’s Hospital, Gothenburg, Rondvägen 10, Gothenburg, 416 85, Sweden

**Keywords:** Crohn’s disease, ulcerative colitis, epidemiology

## Abstract

**Background and Aims:**

Early life may be a critical period for the development of inflammatory bowel disease (IBD) susceptibility. The aim was to examine the association between early-life adherence to a healthy lifestyle and later IBD risk.

**Methods:**

In two Scandinavian birth cohorts (1997-2009), we developed the a priori Modifiable Health-related Lifestyle Score (MHLS) and an empirically weighted Optimal Risk Score (ORS) by ages 1 and 3 years, incorporating data on smoking exposure, delivery mode, breastfeeding, diet quality, antibiotic use, physical activity, and body mass index. Linked national registers identified diagnoses of any IBD, Crohn’s disease (CD), and ulcerative colitis (UC) through 2023-2024. Cox regression and random-effects models, adjusted for parental IBD and sociodemographic characteristics, were used to estimate pooled hazard ratios (aHRs) across MHLS and ORS tertiles.

**Results:**

By a mean age of 20 years at the end of follow-up, 732 of 117 487 participants were diagnosed with IBD. Children with high vs low MHLS by age 1 year had a reduced risk of later IBD (aHR = 0.77 [95% CI = 0.61-0.98]), particularly CD (aHR = 0.57 [0.38-0.86]). ORS analyses yielded estimates nearly identical to MHLS by age 1 year and showed that high vs low adherence to a healthy lifestyle by age 3 was associated with a reduced risk of IBD (aHR = 0.77 [95% CI = 0.63-0.95]) and CD (aHR = 0.68 [95% CI = 0.49-0.94]), but not of UC. Associations were similar for childhood-onset IBD.

**Conclusion:**

A healthy lifestyle in early childhood was associated with a reduced risk of IBD, particularly CD, suggesting that early lifestyle modifications may help prevent the disease.

## 1. Introduction

Inflammatory bowel disease (IBD) is a chronic immune-mediated disease comprising Crohn’s disease (CD) and ulcerative colitis (UC). Following a rising incidence over the past decades, global rates of IBD are continuing to grow.^1^^,^^2^ The rise in IBD incidence parallels our changing lifestyle and emphasizes the key role of the environment in disease development.^3^ Identifying modifiable lifestyle factors can inform preventive efforts against IBD and mitigate its societal burden.^4^

In adults, adherence to overall healthy lifestyle factors has been inversely associated with later IBD risk,^5–7^ and increased adherence to such habits has been reported to prevent 40%-60% of IBD cases (previous studies outlined in Table S1).^7^ Lifestyle habits and potential risk factors for IBD may differ between children and adults. Early-life exposures shape the development of the gut microbiota and immune system and may have lasting effects on IBD risk.^3^ However, no studies have examined the combined impact of health-related, modifiable lifestyle factors in early childhood on subsequent IBD risk or assessed their overall contribution to population-level IBD risk.

Using prospectively collected data on multiple lifestyle factors for IBD, this bi-national cohort study examined whether children adhering to modifiable health-related lifestyle factors by ages 1 and 3 years influence the likelihood of developing IBD.

## 2. Materials and methods

### 2.1. Study population

The study included children from two Scandinavian prospective birth cohorts: All Babies in Southeast Sweden (ABIS) and The Norwegian Mother, Father and Child Cohort Study (MoBa).^8–10^ Briefly, ABIS invited all parents of children born between October 1997 and October 1999 in Southeast Sweden to participate, with 17 055 children included (79% participation rate). MoBa recruited pregnant women across all of Norway, including 114 500 children born in 1999-2009 (41% participation rate). Both cohorts have been followed since pregnancy or birth, using repeated questionnaires to assess the children’s upbringing, lifestyles, health, and socioeconomic status.

**Figure 1.**
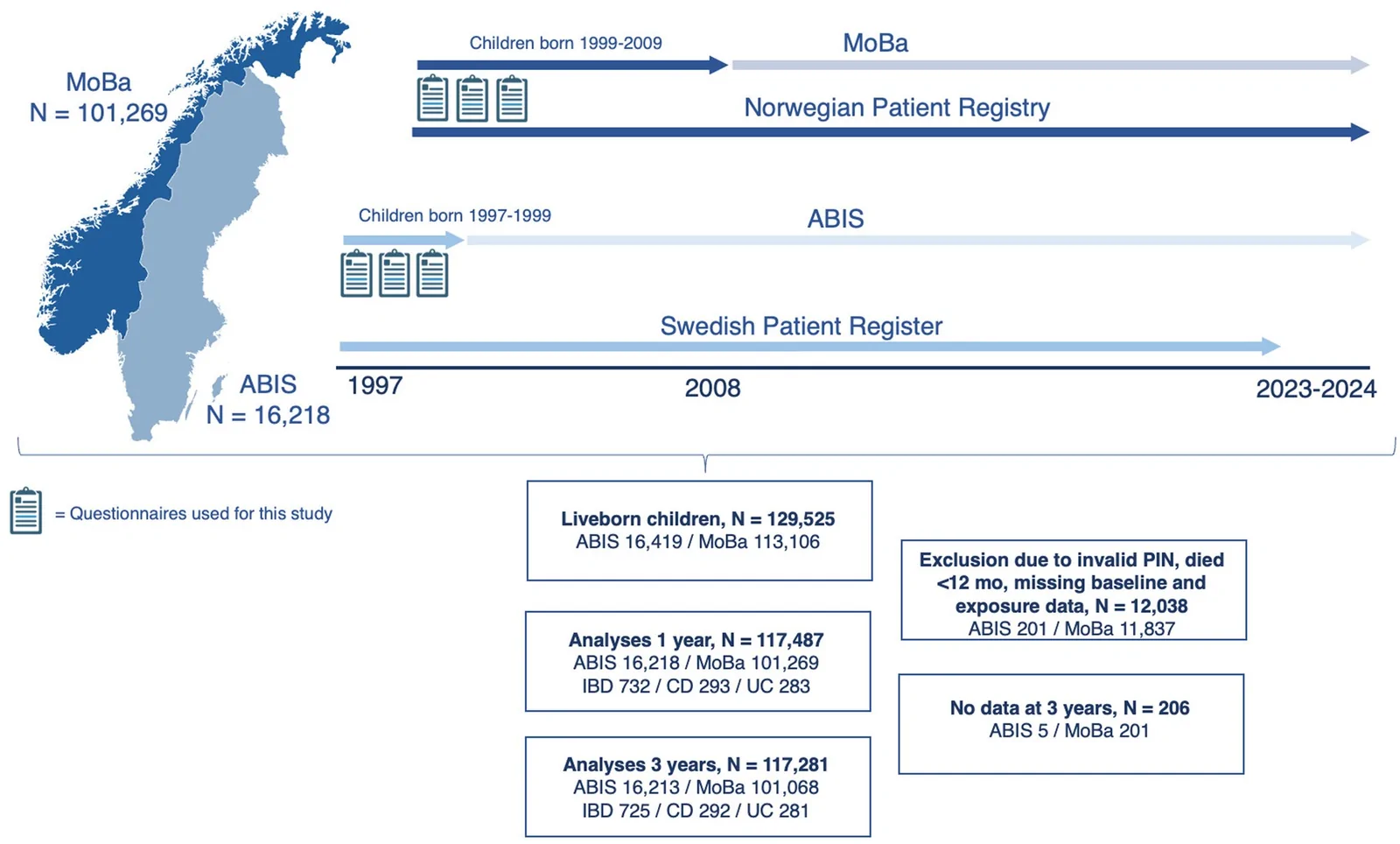
Study flowchart of the ABIS and MoBa cohorts. Data for ABIS were acquired at birth, and at 12 and 30-36 months of age. Data collection in MoBa occurred at gestational weeks 15 and 30, and at 6, 18, and 36 months of age. Children born in 1997-2009 in ABIS and MoBa are included in this study, which uses diagnostic data for inflammatory bowel disease (IBD) linked to personal identity number captured until December 31, 2023 and 2024 from national patient registers. The Norwegian Patient Registry includes register data linked to personal identity numbers from 2008 onwards. IBD also includes IBD-unclassified (1 year, *n* = 156; 3 years, *n* = 152), in similar proportion to previous register-based studies.^63^ The presented numbers represent the mean values across the imputed datasets. Abbreviations: ABIS, All Babies in Southeast Sweden; CD, Crohn’s disease; MoBa, the Norwegian Mother, Father and Child Cohort Study; PIN, personal identity number; UC, ulcerative colitis.

The inclusion criteria of this study were a valid personal identity number to allow linkage to national health registries in Sweden and Norway,^11^^,^^12^ data from enrollment questionnaires, and information on at least one modifiable lifestyle factor, as described below (Figure 1). Registers were linked using unique personal identity numbers, enabling deterministic record linkage (linkage success rate 100%).^13^

### 2.2. Inflammatory bowel disease

We defined IBD using International Classification of Diseases (ICD)-10 codes for the disease in the Swedish and Norwegian national patient registers captured until December 31, 2023 for ABIS and December 31, 2024 for MoBa (Table S2).^14–16^ The algorithm used to identify IBD has demonstrated a positive predictive value of 93%-95% for a clinical IBD diagnosis in Sweden (the ABIS cohort) and Norway in children^17^^,^^18^ and adults.^19^ When validated in a Swedish pediatric population, the algorithm showed an estimated sensitivity of 88% and a specificity of 100%.^20^ Subtype-specific codes were used to identify CD and UC, separately, and the outcome of any IBD also included cases of IBD subtype undetermined by coding, which was defined as individuals with ICD code K52.3 (IBD-unclassified) or those with a mix of codes during the final 2 years of follow-up.

### 2.3. Modifiable health-related lifestyle factors

Similar to previous studies,^5–7^ we pre-selected modifiable health-related lifestyle factors in early childhood that have individually been associated with IBD.^3^^,^^21^ Variables included maternal smoking in pregnancy (no smoking, 1-5 cigarettes/day, >6 cigarettes/day),^22^ delivery mode (vaginal/cesarean section), exclusive breastfeeding duration (<4, 4–5, ≥6 months), passive smoking exposure (yes or no defined by exposure to household smoking by 1 year),^22^ level of diet quality (low, medium, and high defined by a modified Healthy Eating Index),^23^ and antibiotic use by age 1 year (yes/no),^24^ and level of sedentary behavior (<1 and ≥1 h/day defined as screen-time),^25^ physical activity (≥2 and <2 defined as outdoor activity),^25^ and body mass index (BMI, underweight, normal weight, overweight/obese defined by age- and sex-specific cutoffs from the International Obesity Task Force classification^26^) by age 3 years^27^ and are further described in Table S3. The categorizations of health-related lifestyle factors were determined by data availability, questionnaire structure, and the need to harmonize exposure definitions across cohorts.

Data were prospectively collected from a 0- to 12-month diary or from parental-reported questionnaires administered at birth and at child’s age 12 and 30–36 months in ABIS. In MoBa, data were retrieved from questionnaires administered during weeks 15 and 30 of pregnancy, and when the child was 6, 18, and 36 months old (Table S3). The children’s adherence to modifiable health-related lifestyle factors by ages 1 and 3 years was evaluated using two complementary approaches.

Similar to previous studies,^5^^,^^7^^,^^28^ the Modifiable Health-related Lifestyle Score (MHLS) assigned pre-defined weights to each pre-selected factor based on its presumed relevance to IBD risk. For MHLS by 1 year of age, we included the amount of maternal smoking during pregnancy, delivery mode, exclusive breastfeeding duration, exposure to passive smoking, diet quality, and antibiotic use in the first year of life. Mode of delivery was included in the 1-year MHLS because of its effect on the infant gut microbiome composition.^29^ However, because this influence is less prominent after 1 year,^29^ it was omitted from the 3-year MHLS.

By 3 years of age, MHLS also included sedentary behavior, physical activity, and BMI. Based on the literature on IBD risk,^21–25^^,^^27^^,^^30^ and previous studies assessing pre-defined scores of a healthy lifestyle for adult IBD,^5–7^ we a priori selected lifestyle factors to be included in the score. Each lifestyle factor was scored 0-1, with 1 point assigned for each healthy criterion met and 0 for unfavorable exposure. If three levels were available, eg, low, medium, and high diet quality, the medium level was scored 0.5 points. A more detailed description is provided in Table S3. The total MHLS was calculated as the sum of the individual scores and ranged from 0 to 6 points by age 1 year and from 0 to 7 points by age 3 years, with higher scores indicating more favorable lifestyle factors. MHLS was calculated for each cohort separately. Since thresholds defining a healthy lifestyle using lifestyle scores are currently lacking, MHLS was analyzed both as a continuous variable (per-unit increase) and as a trichotomous variable divided by cohort-specific tertiles into low, medium, and high adherence to a healthy lifestyle.

To complement the MHLS, which uses pre-defined scores, we derived an empirically weighted Optimal Risk Score (ORS) at ages 1 and 3 years.^31^ All lifestyle factors included in the MHLS were considered for inclusion in the ORS and were entered jointly into the model using the same categorizations as those used for the MHLS. Maternal smoking during pregnancy was not retained in the final ORS because of its correlation with passive smoking during the child’s first year of life and its non-significant inverse association with IBD risk in the ABIS cohort. The ORS was derived by first fitting a multivariable Cox regression model for IBD and subsequently applying a common scaling factor to the regression coefficients so that the resulting score ranged from 0 to 100, with higher values indicating a greater predicted risk of IBD. The ORS was developed in the ABIS cohort and subsequently applied to both ABIS and the independent MoBa cohort to assess the risks of IBD, CD, and UC. Results were subsequently pooled across cohorts. The development of the ORS within the ABIS cohort was motivated by its higher participation rate and lower level of missing data.

Among the evaluated lifestyle factors, passive smoking and antibiotic use contributed most to both 1- and 3-year ORS (Table S4). The ORS was calculated by summing the weighted factor scores and ranged from 0 to 100, with higher values indicating a greater predicted risk of developing IBD. To ensure comparability with the MHLS, where higher values represent healthier lifestyles, the ORS was analyzed both as a continuous variable per 10-unit decrease (representing improved adherence to health-related lifestyle factors) and as a categorical variable divided into tertiles reflecting low, medium, and high adherence to a healthy lifestyle. Consequently, high adherence corresponded to the lowest tertile of ORS values, representing the lowest predicted risk of IBD.

### 2.4. Covariates

Potential confounders were selected a priori based on a directed acyclic graph (Figure S1) and included variables associated with health behaviors and/or IBD risk.^3^^,^^32^^,^^33^ Covariates included the child’s sex, birth year, parental IBD and country of origin, maternal education level, immune-mediated comorbidities, and age at delivery. Sensitivity analyses were adjusted for the duration of breastfeeding (ie, exclusive breastfeeding). All covariates are defined in detail in Table S3. Data were collected from questionnaires and register data, as detailed in Table S5, and categorized as outlined in Table 1.

**Table 1.** Study characteristics of ABIS and MoBa participants by the Modifiable Health-related Lifestyle Score (MHLS).

	Low	ABIS Medium	High	Low	MoBa Medium	High
**Total *n***	5560	7694	2964	40 297	27 428	33 544
**IBD events, *n* (%)**	61 (1.1)	72 (0.9)	19 (0.6)	265 (0.7)	159 (0.6)	156 (0.5)
**Median follow-up in years (IQR)**	24 (24, 25)	24 (24, 26)	24 (24, 25)	19 (17, 24)	18 (17, 24)	18 (16, 24)
**Median calendar year of birth (range)**	1998 (1997, 1999)	1998 (1997, 1999)	1998 (1997, 1999)	2005 (1999, 2009)	2005 (1999, 2009)	2006 (1999, 2009)
**Sex (%)**						
** Female**	2622 (47.2)	3705 (48.2)	1491 (50.3)	18 999 (47.1)	13 353 (48.7)	17 047 (50.8)
** Male**	2938 (52.8)	3989 (51.8)	1473 (49.7)	21 298 (52.9)	14 075 (51.3)	16 497 (49.2)
**Parental IBD (%)**						
** Yes**	67 (1.2)	91 (1.2)	37 (1.2)	929 (2.3)	584 (2.1)	812 (2.4)
** No**	5493 (98.8)	7603 (98.8)	2927 (98.8)	39 368 (97.7)	26 844 (97.9)	32 732 (97.6)
**Parental country of birth (%)**						
** Sweden/Norway**	4659 (83.8)	6799 (88.4)	2679 (90.4)	34 665 (86.0)	23 120 (84.3)	28 818 (85.9)
** Other**	708 (12.7)	756 (9.8)	236 (8.0)	3809 (9.5)	3113 (11.3)	3424 (10.2)
** Missing**	193 (3.5)	139 (1.8)	49 (1.7)	1823 (4.5)	1195 (4.4)	1302 (3.9)
**Maternal education level (%)**						
** 9-11 years**	804 (14.5)	432 (5.6)	121 (4.1)	4838 (12.0)	2121 (7.7)	1070 (3.2)
** 12 years**	3150 (56.7)	4170 (54.2)	1489 (50.2)	14 525 (36.0)	8234 (30.0)	6976 (20.8)
** ≥13 years**	1421 (25.6)	2947 (38.3)	1301 (43.9)	20 740 (51.5)	16 909 (61.6)	25 347 (75.6)
** Missing**	185 (3.3)	145 (1.9)	53 (1.8)	194 (0.5)	164 (0.6)	151 (0.5)
**Maternal comorbidities (%)**						
** Yes**	249 (4.5)	218 (2.8)	101 (3.4)	1825 (4.5)	1060 (3.9)	1187 (3.5)
** No**	5311 (95.5)	7476 (97.2)	2863 (96.6)	38 472 (95.5)	26 368 (96.1)	32 357 (96.5)
**Maternal age (%)**						
** <25 years**	1130 (20.3)	1104 (14.3)	316 (10.7)	5728 (14.2)	3236 (11.8)	2107 (6.3)
** 25-34 years**	3654 (65.7)	5549 (72.1)	2234 (75.4)	27 660 (68.6)	19 703 (71.8)	25 067 (74.7)
** >35 years**	700 (12.6)	928 (12.1)	353 (11.9)	6883 (17.1)	4475 (16.3)	6356 (18.9)
** Missing**	76 (1.4)	113 (1.5)	61 (2.1)	26 (0.1)	14 (0.1)	14 (0.0)

Descriptive data are presented as numbers (percentages) unless otherwise stated and represent the mean values across the multiply imputed datasets. Definitions of covariates and exposures are provided in Table S5. Maternal comorbidities include type 1 diabetes, thyroid disease, and rheumatoid arthritis.

Abbreviations: ABIS, All Babies in Southeast Sweden; IBD, inflammatory bowel disease; IQR, interquartile range; MoBa, The Norwegian Mother, Father and Child Cohort Study.

### 2.5. Statistical analyses

Cox regression was used to estimate cohort-specific adjusted hazard ratios (aHRs) with 95% confidence intervals (CIs) for the associations of MHLS and ORS with the risk of IBD, CD, and UC. Both scores were analyzed as categorical variables grouped into low, medium, and high categories and as continuous variables (per 1-point increase in score for the MHLS; per 10-point decrease in score for the ORS). The analyses were adjusted for the covariates described above. Follow-up began at the time of exposure assessment (ie, at 1 or 3 years of age) and ended at the time of IBD diagnosis, death, or the end of data capture (December 31, 2023, for ABIS; December 31, 2024, for MoBa), whichever occurred first.

The proportional-hazard assumption was evaluated using the Schoenfeld residuals in complete-case data and was satisfied for all outcomes. Cohort-specific estimates were pooled using the DerSimonian–Laird random-effects method.^34^ The pooled HRs were considered our main results. Between-cohort heterogeneity was assessed using the Cochrane Q and I^2^ statistics for MHLS analyses.^35^ Because the pooled analyses were based on only two cohorts, estimation of between-study variance is subject to considerable uncertainty and was foremost interpreted descriptively.^36^ Multiple imputation by chained equations was employed to address missing data on exposures and covariates, assuming that data were missing at random given the observed covariates. Following previous studies,^7^ we calculated population-attributable risks (PARs) for IBD for the combined factors derived from the pooled aHRs of MHLS and ORS.^37^^,^^38^ Assuming a causal relationship, the PAR represents the proportion of IBD cases that could have been prevented through a population shift to the highest level of adherence to modifiable health-related lifestyle factors.

To assess the robustness of our findings, several sensitivity analyses were conducted. Analyses were performed on complete cases, in addition to those using imputed data. Given that vaginal delivery may be unfavorable under certain medical circumstances, delivery mode was excluded from the 1-year MHLS analyses. To address potential hereditary influences on the association between a healthy lifestyle and IBD risk, we adjusted for parental IBD in the main analyses and additionally examined the interaction between parental IBD and the 1-year MHLS. Since the 3-year MHLS did not include breastfeeding as a variable, these models were additionally adjusted for breastfeeding duration. Finally, we repeated the main analyses limited to childhood-onset IBD (<18 years) and after excluding individuals with very-early onset IBD (<6 years).

In post-hoc analyses, we excluded antibiotics from the 1-year MHLS, as at the individual level, minimization of antibiotic use may not necessarily reflect a health-related lifestyle factor, and repeated the main analyses for IBD and CD risk. We also adjusted the 1-year MHLS analyses for parental household income. Finally, we calculated E-values to estimate the strength of unmeasured confounding required to fully explain the observed observations.^39^ Statistical analyses were conducted using R software (v.4.2.2 and 4.5.1; R Foundation for Statistical Computing, Vienna, Austria).

## 3. Results

### 3.1. Characteristics

Over 2 257 250 person-years of follow-up, 732 IBD events were documented (CD, *n* = 293; UC, *n* = 283; IBD-U, *n* = 156) among the 117 487 children (49% females) included in the analyses of MHLS by 1 year (Figure 1; Table 1). In the cohort-specific analyses, the number of IBD, CD, and UC events were as follows: 152, 56, and 86, in ABIS and 580, 237, and 197 in MoBa. The 3-year MHLS analyses encompassed 117 281 children, resulting in 725 IBD events (CD, *n* = 292; UC, *n* = 281). From age 1 year, children were followed for a median of 24 years in ABIS and 18 years in MoBa, with an overall mean age at the end of follow-up of 20 years. Median ages at diagnosis were 18.7 (interquartile range [IQR], 16.0-21.9 [median age of end of follow-up 25.3 years]) and 15.6 years (IQR, 12.5-17.9 years [median age of end of follow-up 19.3 years]) in ABIS and MoBa, respectively. Most children had a mother with higher education, particularly in MoBa, where a majority had pursued ≥13 years of education. Approximately 1%-2% of the children’s parents had IBD, and over 85% were of Swedish or Norwegian descent. Study characteristics and the distribution of modifiable health-related lifestyle factors among complete cases are presented in Tables S6 and S7.

### 3.2. Modifiable health-related lifestyle factors by 1 year

**Figure 2.**
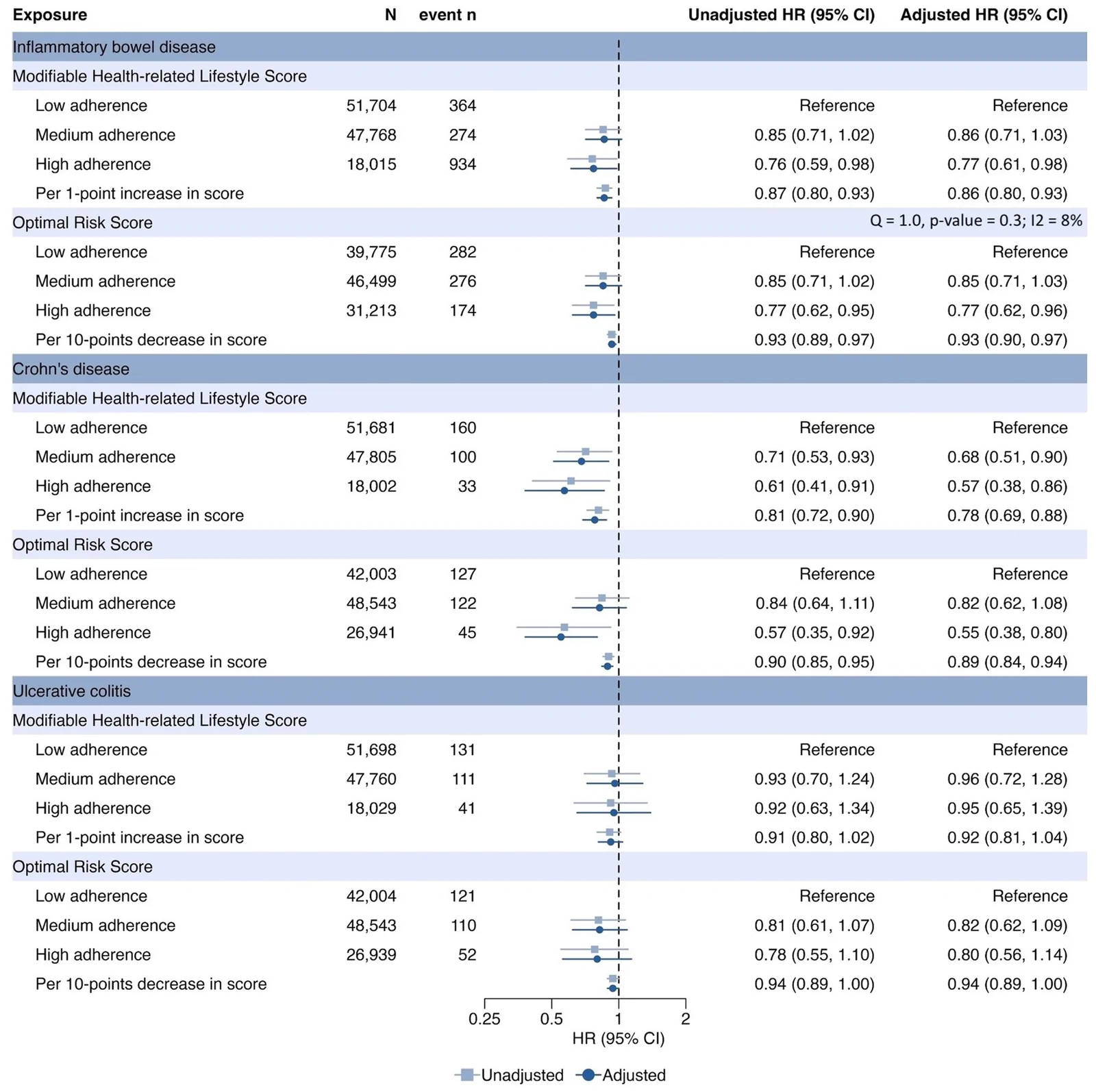
Pooled hazard ratios (HRs) for the Modifiable Health-related Lifestyle Score (MHLS) and the Optimal Risk Score (ORS) by 1 year of age relative to the risk of inflammatory bowel disease, Crohn’s disease, and ulcerative colitis. The HRs were estimated using Cox regression, with missing data handled via multiple imputation. The number of participants (*N*) and events (*n*) represent the mean values across the imputed datasets. IBD overall includes cases classified as IBD-unclassified (*n* = 156). “Low,” “medium,” and “high” correspond to tertiles of adherence to health-related factors of MHLS and ORS (Table S4), where “high” represents the group with the healthiest lifestyle. The MHLS ranges from 0 to 6, with higher values indicating greater adherence to health-related factors. The ORS ranges from 0 to 100, with higher values indicating a higher predicted risk of IBD; thus, lower ORS values correspond to a healthier lifestyle. The ORS analysis was developed on ABIS and applied to both ABIS and MoBa (independent cohort). Analyses were adjusted for the child’s sex, birth year, parental IBD, country of origin, maternal education level, maternal comorbidities, and maternal age at delivery (Table S5). Abbreviations: ABIS, All Babies in Southeast Sweden; MoBa, the Norwegian Mother, Father and Child Cohort Study; CI, confidence interval.

In the pooled analyses of MHLS by 1 year, high vs low adherence to modifiable health-related lifestyle factors was associated with a reduced risk of later IBD when adjusting for the child’s sex, birth year, parental IBD, country of origin, maternal education level, maternal comorbidities, and maternal age at delivery (aHR: 0.77; 95% CI: 0.61-0.98; Q = 1.0, *P* = .3; I^2^ = 8%; Figure 2). Each 1-point increase in MHLS by 1 year was associated with an aHR of 0.86 (95% CI: 0.80-0.93) for IBD when analyzed as a continuous variable. The association was driven by CD (medium vs low MHLS, aHR: 0.68; 95% CI: 0.51-0.90; high vs low MHLS, aHR: 0.57; 95% CI: 0.38-0.86; Figure 2). The aHR per 1-point increase in MHLS by 1 year was 0.78 (95% CI: 0.69-0.88) for CD. No association was observed between high vs low MHLS by 1 year and later UC risk (aHR: 0.95; 95% CI: 0.65-1.39; per 1-point increase in score, aHR: 0.92; 95% CI: 0.91-1.04; Figure 2).

Consistent with the MHLS associations, pooled ORS analyses of ABIS and MoBa showed that children with a greater adherence to modifiable health-related lifestyle factors by 1 year of age had a reduced risk of IBD (aHR: 0.77; 95% CI: 0.62-0.96). Comparable to the results for MHLS, high vs low ORS by 1 year of age were associated with a reduced risk of CD (aHR: 0.55; 95% CI: 0.38-0.80), but not with UC (Figure 2).

### 3.3. Modifiable health-related lifestyle factors by 3 years

**Figure 3.**
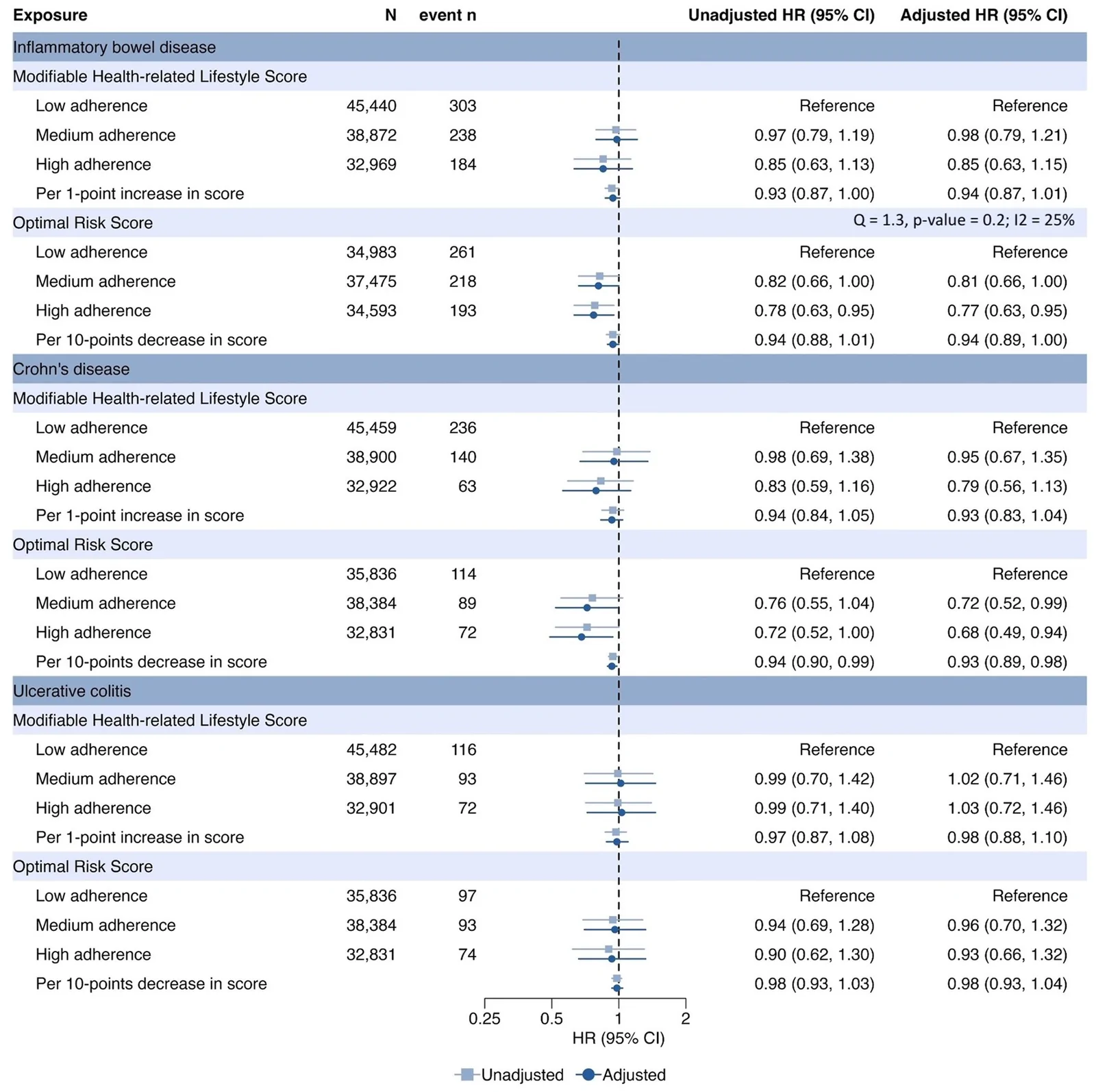
Pooled hazard ratios (HRs) for the Modifiable Health-related Lifestyle Score (MHLS) and the Optimal Risk Score (ORS) by 3 years of age in relation to the risk of inflammatory bowel disease, Crohn’s disease, and ulcerative colitis. The HRs were estimated using Cox regression, with missing data handled via multiple imputation. The number of participants (*N*) and events (*n*) represent the mean values across the imputed datasets. IBD overall includes cases classified as IBD-unclassified (*n* = 156). “Low,” “medium,” and “high” correspond to tertiles of adherence to health-related factors of MHLS and ORS (Table S4), where “high” represents the group with the healthiest lifestyle. The MHLS ranges from 0 to 6, with higher values indicating greater adherence to health-related factors. The ORS ranges from 0 to 100, with higher values indicating a higher predicted risk of IBD; thus, lower ORS values correspond to a healthier lifestyle. The ORS analysis was developed on ABIS and applied to both ABIS and MoBa (independent cohort). Analyses were adjusted for the child’s sex, birth year, parental IBD, country of origin, maternal education level, maternal comorbidities, and maternal age at delivery (Table S5). Abbreviations: ABIS, All Babies in Southeast Sweden; CD, Crohn’s disease; MoBa, the Norwegian Mother, Father and Child Cohort Study; CI, confidence interval.

In the pooled 3-year MHLS analyses, pooled aHRs for IBD were 0.98 (95% CI: 0.79-1.21; Figure 3) and 0.85 (95% CI: 0.63-1.15; Q = 1.3, *P* = .2; I^2^ = 25%) for medium and high versus low adherence to modifiable health-related lifestyle factors, respectively; each point increase in MHLS was associated with an aHR of 0.94 (95% CI: 0.87-1.01). For CD, medium and high vs low MHLS yielded a pooled aHR of 0.95 (95% CI: 0.67-1.35) and 0.79 (95% CI: 0.56-1.13; Figure 3), respectively (per 1-point increase aHR: 0.93; 95% CI: 0.83-1.04). Pooled analyses across ABIS and MoBa showed that high vs low ORS by 3 years was associated with a reduced risk of IBD (aHR: 0.77; 95% CI: 0.63-0.95), and CD (aHR: 0.68; 95% CI: 0.49-0.94; MHLS, per 1-point increase aHR: 0.93; 95% CI: 0.89-0.98). Neither the MHLS nor the ORS analyses showed an association between adherence to a health-related lifestyle by age 3 years and later UC risk.

### 3.4. Population attributable risk

Assuming a causal relationship at the population level, an increased adherence from low and moderate to high MHLS by age 1 year could have prevented 32% (95% CI: 10%-50%) of CD cases. The corresponding PAR estimate defined by 1-year ORS was 38% (95% CI: 0%-62%) for CD (Table S8). By 3 years, PAR for CD was 18% (95% CI: −1% to 35%) when defined by MHLS and 14% (95% CI: −9% to 33%) when determined by ORS. For any IBD, PAR ranged from 14% to 20% by 1 year of age (ORS, PAR: 14%; 95% CI: 1%-36%; MHLS, PAR: 20%; 95% CI: −7% to 41%), and from 8% to 9% (ORS, PAR: 8%; 95% CI: 4%-20%; MHLS, PAR: 9%; 95% CI: −5% to 22%) by 3 years of age. The PAR for UC by 1 and 3 years was low and non-significant (Table S8).

### 3.5. Cohort-specific analyses

Cohort-specific estimates are presented in Tables S9 and S10. For the continuous 1-year MHLS, the aHR for IBD was 0.81 (95% CI, 0.61-1.07) in ABIS and 0.86 (95% CI, 0.78-0.96) in MoBa. Corresponding estimates for the 3-year MHLS were 0.94 (95% CI, 0.73-1.19) and 0.92 (95% CI, 0.82-1.03), respectively (Table S9). For the continuous ORS, the adjusted aHR per 10-point decrease in score was 0.89 (95% CI, 0.82-0.98) in ABIS and 0.94 (95% CI, 0.90-0.98) in MoBa at 1 year, and 0.90 (95% CI, 0.82-0.98) and 0.96 (95% CI, 0.93-1.00) at 3 years, respectively (Table S10).

### 3.6. Sensitivity analyses

The significant associations between MHLS by 1 year of age and risk of IBD and CD remained unchanged after excluding delivery mode from the score (Table S11) and were not significantly influenced by parental IBD status in ABIS (*P* for interaction = .65) or MoBa (*P* for interaction = .60). The non-significant associations for MHLS by 3 years and IBD, CD, and UC remained when additionally adjusting for exclusive breastfeeding duration (Table S12). The estimates for MHLS remained largely consistent in pooled analyses restricted to childhood-onset IBD and when excluding very early-onset IBD (Table S13).

### 3.7. Posthoc analyses

The associations between high vs low MHLS by 1 year of age and risk of IBD and CD remained unchanged after excluding antibiotics from the score (Table S14). Additional adjustment for parental household income yielded relatively unchanged estimates for MHLS by 1 year and IBD and CD risk (Table S15). E-values calculated from the pooled aHRs for the associations between MHLS and ORS by 1 year and the risks of IBD and CD were all >1.90 (Table S16).

## 4. Discussion

In this bi-national birth cohort study, prospectively recorded data of high adherence to modifiable health-related lifestyle factors by 1 and 3 years of age were associated with a reduced risk of later CD. These findings were consistent after adjusting for potential confounders, including the child’s sex, parental IBD, and sociodemographic factors. No clear associations were observed for high adherence to modifiable health-related lifestyle factors by ages 1 or 3 years and subsequent UC risk. Our data suggest that efforts to promote a healthy lifestyle in early life may be a potential prevention strategy for IBD, particularly CD.

This study is novel due to its use of two large birth cohorts with prospectively collected data to examine the joint associations between lifestyle factors in early life and IBD risk. Few studies have prospectively examined the overall impact of a healthy lifestyle on IBD risk, and existing data are limited to adults.^6^^,^^7^ Sun  et al. used UK Biobank cohort data on five health-related factors (dietary quality, alcohol intake, smoking, sleep, and physical activity). They found that an adverse lifestyle was associated with a 2-fold increase in CD and UC risk.^6^ Another study from the UK Biobank cohort showed that an increased number of healthy lifestyle factors was associated with a reduced risk of IBD, with three to five healthy lifestyle factors reducing the risk by 50% compared to no factors.^5^ Using data from three prospective US cohorts, Lopes and colleagues determined that five unhealthy lifestyle behaviors were associated with a nearly 2-fold increased risk for both CD and UC; these results were subsequently confirmed in a European cohort.^7^ Moreover, their findings indicate that at a population level adherence to low-risk factors might have prevented 43% and 44% of CD and UC cases, respectively.^7^

We found that high adherence to modifiable health-related lifestyle factors by 1 and 3 years of age was associated with reduced CD risk, with the strongest association observed in the 1-year analyses; in pooled analyses, high vs low adherence to MHLS by age 1 year was associated with a 23% lower risk of IBD (aHR: 0.77; 95% CI: 0.61-0.98) and 43% lower risk of CD (aHR: 0.57; 95% CI: 0.38-0.86). While non-casual explanations cannot be ruled out, these observations suggest that early life, even in the first year, may have lasting effects on later IBD risk, particularly CD. One possible explanation relates to the development of the gut microbiome, which is more vulnerable to environmental factors in the first year of life compared to at 3 years,^34^ as well as maturation of the immune system, which seems particular sensitive to environmental exposures during the first 2 years of life.^40^ The lack of associations for UC may reflect etiological differences between CD and UC. Individuals genetically predisposed to CD appear to be more susceptible to reduced microbial diversity, whereas dysbiosis in UC has been observed to be generally milder and has been suggested to arise more from mucosal immune dysregulation than from primary alterations in the gut microbiome.^41^ While more research is needed, data from adults indicate distinct microbiome–lifestyle interactions between IBD subtypes.^41^

Consistent with adult data indicating that healthy habits may help prevent IBD, our findings further underscore the importance of adopting lifestyle modifications early. Interventions that focus on lifestyle choices during childhood may be especially beneficial, given the tendency for early-formed behaviors to endure.^42^ While no preventive approach for IBD exists,^7^ there are ongoing prevention trials targeting individuals at high risk for IBD.^43^^,^^44^ In the general population, modifiable lifestyle factors are attractive preventive targets, as efforts promoting these behaviors entail minimal risk and can benefit multiple health domains,^28^^,^^45^^,^^46^ including other immune-mediated diseases.^47–50^ Healthy lifestyle habits, including diet and physical activity, are in adults widely recognized as key preventive strategies for non-communicable diseases at the individual level.^51^ This suggests that preventive strategies promoting healthy behaviors early in life may yield broad health benefits across multiple disease areas. Importantly, a lifestyle-based intervention carries a relative low cost compared to more invasive, pharmacological strategies for disease prevention.^52^ In light of the anticipated rise in global prevalence,^53^ delaying onset or preventing even a small percentage of cases would considerably benefit individuals, healthcare systems, and society.

### 4.1. Strengths and limitations

A key strength of this study is the use of data from two prospective birth cohorts with comparable study characteristics and long-term follow-up through national patient register linkage. We also examined the consistency of our findings across the pre-specified MHLS and the empirically weighted ORS methods used for modeling health habits, including exclusive breastfeeding^54^ and dietary habits that have been associated with IBD risk.^55^ Our findings were consistent across multiple sensitivity analyses, including complete-case analyses, restriction to childhood-onset IBD, exclusion of very early-onset IBD, exclusion of antibiotics from the 1-year MHLS, and with additional adjustment for parental household income. The availability of detailed questionnaire data and linked register information allowed adjustment for a range of potential confounders, including child and parental characteristics, including IBD heredity (maternal or paternal IBD).

Our prospective data collection probably improved the precision and granularity of exposure assessment, which is vital for IBD, given the suspected long latency period between exposure and disease onset. With median follow-up durations of 18 (MoBa) to 24 (ABIS) years, we identified IBD cases across adolescence and early adulthood, a period coinciding with a particularly high incidence of IBD.^56^ A register-based outcome algorithm with a positive predictive value (PPV) of ≥93% for IBD in Sweden and Norway^17^^,^^18^^,^^20^ was implemented in both cohorts. More specifically, this included validation in the ABIS cohort,^17^ as well as external validation in other Swedish populations.^19^^,^^20^ We acknowledge that the generalizability of PPVs estimated in external validation studies should be considered and that PPVs depend on the true prevalence, sensitivity, and specificity of the condition being tested.^57^^,^^58^ We also acknowledge that we cannot rule out the risk of misclassification for IBD, particularly given the lower PPV for specific IBD subtypes.^18^^,^^20^ The proportion of IBD-U events in our study population (21%) may be explained by the high proportion of recently diagnosed patients with relatively few diagnostic listings at the end of data capture. However, similar proportions have been reported in other studies in Sweden^59^ and across European countries.^60^

We acknowledge that our definition of low, medium, and high adherence to a healthy lifestyle was pragmatically defined, similar to previous studies,^5^^,^^7^^,^^28^ and that additional modifiable lifestyle factors may influence IBD development. For example, we did not include psychological factors such as parental stress, which have been less well examined in relation to IBD risk.^61^ The definition of modifiable health-related lifestyle factors is relatively broad and includes exposures that may be amenable to intervention at either the individual or population level, even though modification may not be appropriate in all circumstances. For example, antibiotic exposure may increase IBD risk at the population level but remain necessary when clinically indicated. The favorable influence from modifiable lifestyle factors may reflect the clustering of healthy behaviors; for example, children whose mothers did not smoke during pregnancy are more likely to have healthier diets and higher physical activity levels than those whose mothers who smoked. Self-selection for participation could reduce variation in lifestyle exposures within our sample, thereby reducing external validity. Nevertheless, previous analyses from MoBa have shown that such differences have not affected exposure–outcome associations.^62^ We acknowledge that there is a risk of erroneous recall of exposures when relying on self-reported questionnaires. However, information on several lifestyle habits, including diet quality and physical activity, is not available in registers.

As in any large-scale birth cohort study with long-term follow-up, missing data are inevitable, and the potential impact on our findings is unknown; unmeasured health-related factors, including rural living, air pollution, green space, blue space, water quality, or other potential residual confounders, may have influenced the observed associations. However, estimated E-values were all >1.90, suggesting that the observed association would need to be influenced by at least moderately strong unmeasured confounding to fully explain the results. We also acknowledge that variation in questionnaire wording and timing limited the level of detail for some lifestyle factors and restricted our ability to analyze all lifestyle factors as continuous variables. To address cohort heterogeneity, we used a random-effects model to pool analyses and presented cohort-specific analyses. Our study pertained to IBD diagnosed in children and young adults, and the influence of healthy lifestyle habits on IBD risk later in life could not be examined. While there is no validated tool to measure a healthy lifestyle in children, our approach builds on adult IBD studies assessing the combined effects of healthy lifestyle factors.^5–7^ We also acknowledge that the MHLS assumes equal contributions of each component to IBD risk, whereas the ORS may be susceptible to overfitting because it was derived within a subset of the study population (ABIS). In addition, because the ORS was developed using overall IBD rather than CD and UC separately, associations between lifestyle factors and the risks of CD and UC may be attenuated. These considerations motivated the use of two complementary approaches to measure the joint associations for healthy lifestyle factors. Importantly, the similarity of results across MHLS and ORS analyses supports the overall interpretation of the findings.

There are known homogeneities within the study population, such as a high level of maternal education and a low maternal smoking rate.^62^ Thus, our population-based bi-national cohort design can be applied to children with comparable lifestyle habits. However, the findings of our study may not be fully generalizable to populations with different characteristics, including higher levels of smoking and lower levels of maternal education. Further studies should investigate whether healthy lifestyle patterns in later childhood and across different populations are associated with subsequent IBD risk. While we did not detect an influence on our results from parental IBD status, we could not assess the extent to which genetic susceptibility markers for IBD might influence the observed association with overall health habits, or the extent to which changes in the gut microbiome and immune system may mediate these associations. Lack of prior data on lifestyle factors in young children and IBD limited our ability to define variable weights based on independent cohorts. While our analyses were based on pre-selected lifestyle factors, future studies may explore cluster-based approaches to identify patterns of co-occurring lifestyle behaviors for IBD prevention. Lastly, we cannot rule out the possibility that the observed associations with early-life lifestyle factors may be mediated by later-life lifestyle habits, including smoking.

## 5. Conclusions

This bi-national Scandinavian birth cohort study found that high adherence to modifiable health-related lifestyle factors in early childhood was associated with a lower risk of developing CD. Still, no clear association was observed for later UC. Our data suggest that efforts to adopt healthy lifestyle modifications in early life may be an attractive target for future prevention strategies against IBD, particularly CD.

## Supplementary Material

jjag114_Supplementary_Data

## Data Availability

All relevant data are included in the paper and its supplementary material. The lead authors (the manuscript’s guarantors) affirm that the manuscript is an honest, accurate, and transparent account of the study being reported. Because of participants’ confidentiality and European law, the cohort and register data used in this study cannot be openly distributed. Data from ABIS can be obtained from Johnny Ludvigsson upon reasonable request, provided ethical approval has been received. Researchers can apply for access to the MoBa cohort data and to register data from relevant health authorities.
