# Supplementary material for: Modifiable lifestyle factors in early life and inflammatory bowel disease risk in two birth cohorts
Source: J Crohns Colitis. 2026 Aug 24;20(8):jjag114. doi: 10.1093/ecco-jcc/jjag114 (PMC13503009; doi:10.1093/ecco-jcc/jjag114)
Supplement: Supplementary file 1 [file jjag114_supplementary_data.docx]

SUPPLEMENTAL FILE FOR

Guo et al. Modifiable lifestyle factors in early life and inflammatory bowel disease risk in two birth cohorts

Table of content

[**Supplementary Figure 1.** Directed acyclic graph	2](#_Toc231383781)

[**Supplementary Table 1.** Earlier publications	3](#_Toc231383782)

[**Supplementary Table 2.** International Classification of Diseases (ICD)-10 codes for inflammatory bowel disease^a^	5](#_Toc231383783)

[**Supplementary Table 3**. Description, definition, and scoring of modifiable lifestyle factors included in the Modifiable Health-related Lifestyle Score (MHLS) at 1 and 3 years	6](#_Toc231383784)

[**Supplementary Table 4.** Description of lifestyle factor weights in the Optimal Risk Scoring (ORS) model by 1 and 3 years of age	8](#_Toc231383785)

[**Supplementary Table 5.** Description of covariates and their data sources	9](#_Toc231383786)

[**Supplementary Table 6.** Participant baseline characteristics from the ABIS and MoBa cohorts, by tertiles of the Modifiable Health-related Lifestyle Score (MHLS) by 1 year	10](#_Toc231383787)

[**Supplementary Table 7.** Distribution of modifiable lifestyle factors included in the Modifiable Health-related Lifestyle Score (MHLS)	11](#_Toc231383788)

[**Supplementary Table 8.** Population attributable risk for Modifiable Health-related Lifestyle Score (MHLS) and Optimal Risk Score (ORS) by 1 and 3 years	13](#_Toc231383789)

[**Supplementary Table 9.** Cohort-specific and pooled analyses of the Modifiable Health-related Lifestyle Score (MHLS) by 1 and 3 years of age in complete case analyses.	14](#_Toc231383790)

[**Supplementary Table 10.** Cohort-specific analyses of the Optimal Risk Score (ORS) by 1 and 3 years of age in imputed data	16](#_Toc231383791)

[**Supplemental Table 11.** Pooled adjusted hazard ratios in analyses with and without delivery mode for the Modifiable Health-related Lifestyle Score (MHLS) by 1 year in complete case data	18](#_Toc231383792)

[**Supplementary Table 12.** Pooled adjusted hazard ratios with and without additional adjustment for full breastfeeding duration for the Modifiable Health-related Lifestyle Score (MHLS) at 3 years of age, and risk of IBD, CD, and UC in imputed datasets.	19](#_Toc231383793)

[**Supplementary Table 13.** Pooled adjusted hazard ratios restricted to childhood-onset IBD and excluding those with VEO-IBD for the Modifiable Health-related Lifestyle Score (MHLS) at 1 and 3 years of age, and risk of IBD in imputed datasets.	20](#_Toc231383794)

[**Supplementary Table 14.** Associations between Health-related Lifestyle Score (MHLS) at 1 of age and risk of IBD and CD after excluding antibiotics from the score in imputed datasets	21](#_Toc231383795)

[**Supplementary Table 15.** Associations between Health-related Lifestyle Score (MHLS) at 1 of age and risk of IBD and CD after additional adjustments for parental household income in imputed datasets.	22](#_Toc231383796)

[**Supplementary Table 16.** E-values for associations between MHLS and ORS at 1 year and risk of IBD and CD, based on pooled analyses across the ABIS and MoBa cohorts.	23](#_Toc231383797)

[**Supplementary Table 17.** The RECORD statement checklist	24](#_Toc231383798)

**Supplementary Figure 1.** Directed acyclic graph illustrating the hypothesised relationships between parental IBD,^1^ maternal age at delivery,^2^ maternal comorbidities,^3, 4^ maternal education level,^5^ parental origin of birth,^6^ calendar year of birth, and child’s sex^7^ and early-life modifiable health-related lifestyle factors and IBD risk, with exposure (green), outcome (blue), potential confounders (red), external variables (white). IBD, inflammatory bowel disease.

**Supplementary Table 1.** Earlier publications on adult healthy lifestyle factors and inflammatory bowel disease risk

| Reference | Country | Study design | Study population | Exposure | Scale of assessment | Outcome (events) | Results (95% CI) |
| --- | --- | --- | --- | --- | --- | --- | --- |
| Ho et al. 2025^8^ | United Kingdom | Prospective cohort study based on the UK Biobank.  Recruiting from 2006 to 2010. | Aged 37–73 years. | Healthy lifestyle behaviours include never smoking, optimal sleep, high levels of vigorous physical activity, high dietary quality, and moderate alcohol intake.  *Dietary Approaches to Stop Hypertension (DASH) diet | Total score 0, 1, 2, or 3–5. | 514 IBD  173 CD  373 UC | Adhering to 1, 2, and 3–5 behaviors: adjusted HRs (95% CI) compared to none:  IBD: 0.75 (0.59–0.97), 0.72 (0.56–0.92), and 0.50 (0.37–0.68), respectively.  CD: 0.66 (0.43–1.03), 0.76 (0.49–1.16), 0.50 (0.29–0.84), respectively.  UC: 0.75 (0.56–0.99), 0.64 (0.48–0.86), 0.48 (0.34–0.69), respectively. |
| Sun et al. 2023^9^ | United Kingdom | Prospective cohort study based on the UK Biobank.  Recruiting from 2006 to 2010. | European ancestry.  Mean age 56.7 (8.0).  Median age of diagnosis CD, 65 years (range: 43–82) and UC, 66 years (range: 43–82). | Modifiable Health-related Lifestyle Score and genetic risk. | Favorable (4 or 5 healthy lifestyle factors), intermediate (3 healthy lifestyle factors), and unfavorable (0–2 healthy lifestyle factors) lifestyles.  Weighted standardized Modifiable Health-related Lifestyle Score. | 707 CD  1 576 UC | aHR* (95%CI), compared to a favorable healthy lifestyle:  CD,  Intermediate lifestyle,  1.36 (1.13–1.62):  Unfavorable lifestyle,  1.94 (1.61–2.33)  UC,  Intermediate lifestyle,  1.50 (1.34–1.68):  Unfavourable lifestyle,  1.98 (1.73–2.27)  *Adjusted for age, age-square, sex, Townsend deprivation index, education, Charlson comorbidity index, the first 20 principal components of ancestry, and weighted lifestyle category or genetic risk category. |
| Lopes et al. 2022^10^ | United States & Europe | Participants from three prospective cohorts: the Nurses’ Health Study (NHS), Nurses’ Health Study II (NHSII), and Health Professionals Follow-up Study (HPFS).  Validation in three European cohorts: the Swedish  Mammography Cohort (SMC), the Cohort of Swedish Men (CoSM), and the European Prospective Investigation into Cancer and Nutrition (EPIC).  Baseline range 1986–1999. | Females 25–42 years, Males 40–75 years | Modifiable risk score (MRS) based on body mass index, smoking status, non-steroidal anti-inflammatory drug use, physical activity, daily consumption of fruit and vegetables, fiber, n3:n6 polyunsaturated fatty acids, and red meat.  Modifiable Health-related Lifestyle Score (HLS): adherence to recommendations by the United States (US) Department of Health and Human Services and US Department of Agriculture, Dietary Guidelines for Americans, and the American Heart Association Guidelines for Healthy Living.  Population attributable risk (PAR) score. | MRS range 0–6 points.  Low-risk group (reference) defined as score 0–1 for CD, or 0–2 for UC.  HLS range 0–9.  Healthy group (reference) is defined as a score of 7–9, and an unhealthy group is defined as a score <7. | 345 CD  456 UC | Compared to the low-risk group,  CD-MRS >1 p-trend <0.0001;  UC-MRS >2 p-trend 0.0008  Compared to participants with a CD-MRS of 0–1,  CD-MRS of 6 = aHR 4.15 (95%CI: 1.95–8.84)  Compared to those with a UC-MRS of 0–2,  UC-MRS of 6 = aHR 2.78 (95%CI: 1.47–5.25)  Compared to the Healthy group, adherence to the American Healthy lifestyle guidelines  CD: HLS <7 p-trend 0.004  UC: HLS <7 p-trend 0.02  The estimated preventable PAR for CD and UC ranged from 42 to 61% across the three primary cohorts. |

CI, confidence interval; CD, Crohn’s disease; HLS, Modifiable Health-related Lifestyle Score; HR, hazard ratio; IBD, inflammatory bowel disease; MRS, modifiable risk score; PAR, population attributable risk; UC, ulcerative colitis.

## **Supplementary Table 2.** International Classification of Diseases (ICD)-10 codes for inflammatory bowel disease^a^

| **Disease** | **ICD-10 (1997-)** | **Description of outcome** |
| --- | --- | --- |
| Inflammatory bowel disease | K50, K51, K52.3 | Minimum two inpatient or non-primary outpatient care diagnoses. Those with a mix of CD, UC, and IBD-U codes during the past 2 years of follow-up were classified as (“non-specific”) IBD-U.^b^ Date of IBD diagnosis was defined as the first recorded diagnosis. |
| Crohn’s disease | K50 |  |
| Ulcerative colitis | K51 |  |
| IBD-unclassified | K52.3 or a mix of ICD-codes for IBD during the past 2 years of follow-up |  |

^a^Based on a validated register-based IBD definition^11, 12^

^b^IBD-U was not included as a separate outcome and refers in this study to IBD subtype undetermined by coding.

CD, Crohn’s disease; IBD, inflammatory bowel disease; IBD-U, inflammatory bowel disease unclassified; UC, ulcerative colitis.

## **Supplementary Table 3**. Description, definition, and scoring of modifiable lifestyle factors included in the Modifiable Health-related Lifestyle Score (MHLS) at 1 and 3 years

| **Modifiable lifestyle factor** | **Definition and data sources** | **MHLS weight** | **Included in** | **Original study** |
| --- | --- | --- | --- | --- |
| Maternal smoking amount during pregnancy | Mothers were asked to report daily cigarette consumption on the birth questionnaire (ABIS) and at weeks 15 and 30 of pregnancy, as well as when the child was 6 months old (MoBa).^13^ | No smoking = 1  1-5 cigarettes = 0.5  ≥6 cigarettes = 0 | MHLS 1 and 3 years | Sigvardsson et al. 2024. PMID: 38329478. |
| Delivery mode | Mothers reported vaginal delivery or caesarean section in questionnaires administered at birth (ABIS) and 6 months (MoBa). | Vaginal = 1  Caesarean section = 0 | MHLS 1 year |  |
| Exclusive breastfeeding duration | A 1-year parental diary in which mothers were instructed to report the exact date of cessation of any breastfeeding. When diary data were missing, ABIS data on any breastfeeding duration were retrieved from the 12-month questionnaire. Questionnaires were administered at ages 6 and 18 months in MoBa.^14^ | ≥6 months = 1  4–5 months = 0.5  <4 months = 0 | MHLS 1 year | Agrawal et al. 2024. PMID: 38729392 |
| Passive smoking by age one year | At the child’s age of 1 year, parents reported if anyone smoked in the household during the child’s first year of life (ABIS). The MoBa questionnaire collected information about parental smoking habits during the postpartum period, which was administered at 6 months.^13^ | No smoking = 1  Passive smoking = 0 | MHLS 1 and 3 years | Sigvardsson et al. 2024. PMID: 38329478. |
| Diet quality by age one year | Dietary data were obtained from specific food questions in questionnaires administered at 12 and 30–36 months in ABIS and at 18 and 36 months in MoBa. Diet quality was assessed using a modified version of the Healthy Eating Index (HEI), which reflects the child’s overall adherence to dietary recommendations for children. The total HEI was divided into thirds within each cohort, representing low, medium, and high diet quality.^15^ | High = 1  Medium = 0.5  Low = 0 | MHLS 1 and 3 years | Guo et al. 2024. PMID: 38290832 |
| Antibiotic use by age one year | All systemic antibiotic consumption in the first year of life. In MoBa, parents reported the name of any medication used by the child through an ‘open-ended question.’ In ABIS, parents were asked to disclose the name of the medication administered to their child by age 1 year.^16^ | No antibiotic = 1  Any antibiotics course = 0 | MHLS 1 and 3 years | Mårild et al. 2025. PMID: 39450871 |
| Sedentary behaviour by age three years | Sedentary activity, defined as time spent in front of any screen (e.g., computer, television), was collected at the 3-year questionnaire.^17^ | <1 hour/day = 1 ≥1 hour/day = 0 | MHLS 3 years | Lerchova et al. 2023. 37792586 |
| Physical activity by age three years | Physical activity was defined as the time spent on outdoor activities and was assessed using a 3-year questionnaire.^17^ | ≥2 hours/day = 1 <2 hours/day = 0 | MHLS 3 years | Lerchova et al. 2023. 37792586 |
| Body mass index by age three years | Body mass index (kg/m^2^) was calculated using the child’s parental-reported length/height^18^ and weight at 3 years of age. Childhood BMI categories were defined using age- and sex-specific cut-off points for thinness, normal weight, overweight, and obesity derived from the International Obesity Task Force classification ^19^ | Normal weight = 1  Underweight, overweight, obese = 0 | MHLS 3 years | Lerchova et al. 2025. PMID: 40854285 |

Definitions and data sources are provided for each modifiable lifestyle factor. Scores were assigned according to prior literature, with higher values indicating more favourable (healthier) exposures. The total Modifiable Health-related Lifestyle Score (MHLS) was calculated as the sum of the individual exposure scores and ranged from 0 to 6 points at 1 year and from 0 to 7 points at 3 years, with higher scores indicating greater adherence to a healthy lifestyle. The categorization of lifestyle factors was based on available data granularity and distribution, established recommendations, and previous literature. Two-category classifications were used when recommendations supported a binary distinction (e.g. smoking during pregnancy) or when the questionnaire data did not allow a more detailed categorization*.* The categorization of variables was also guided by the need to harmonize exposure definitions across the two cohorts while maintaining sufficient comparability.

Cut-offs for the 1-year MHLS into tertiles were: ABIS, low (≤4.0), medium (=4.5), and high (≥5.0); MoBa, low (≤3.5), medium (4.0–4.5), and high (≥5.0).

Cut-offs for the 3-year MHLS into tertiles were: ABIS, low (≤4.0), medium (4.5–5.0), and high (≥5.5); MoBa, low (≤4.0), medium (4.5–5.0), and high (≥5.5).

ABIS, All Babies in Southeast Sweden; MoBa, the Norwegian Mother, Father and Child Cohort Study.

## **Supplementary Table 4.** Description of lifestyle factor weights in the Optimal Risk Scoring (ORS) model by 1 and 3 years of age

| **Exposure** | **Level** | **ORS weight by 1 year** | **ORS weight by 3 years** |
| --- | --- | --- | --- |
| Breastfeeding duration <5 months |  | 9.2 | - |
| Caesarean section |  | 34.5 | - |
| Antibiotic use in the first year of age |  | 16.8 | 24.5 |
| Diet quality by age 1 year | Low | 6.9 | 9.8 |
|  | Medium | 6.1 | 8.1 |
| Passive smoking by age 1 year |  | 32.6 | 51.5 |
| Underweight, overweight, and obese by age 3 years |  | - | 8.0 |
| Physical activity <2 hours/day by age 3 years |  | - | 2.6 |
| Sedentary behaviour <1 hour/day by age 3 years |  | - | 3.6 |

The Optimal Risk Score (ORS) was derived using a multivariable Cox regression model for IBD in the ABIS cohort. The resulting weights reflect the relative contribution of each lifestyle factor to IBD risk. The regression coefficients were subsequently scaled to produce a total score ranging from 0 to 100, with higher values indicating a greater predicted risk of IBD. Maternal smoking during pregnancy was not retained in the final ORS because of its correlation with passive smoking during the child's first year of life and its non-significant inverse association with IBD risk in the ABIS cohort.

ORS tertile cut-offs were based on the average tertile boundaries across the imputed datasets. Cut-offs for the 1-year ORS were: ABIS, low (≤6.1), medium (6.9–23.7), and high (≥26.0); MoBa, low (≤58.2), medium (58.6–83.9), and high (≥90).

Cut-offs for the 3-year ORS were: ABIS, low (≤17.9), medium (18.0–32.0), and high (>32.0); MoBa, low (≤65.8), medium (67.5–87.7), and high (≥89.4).

Definitions of all exposures are provided in Supplementary Table 3.

## **Supplementary Table 5.** Description of covariates and their data sources

| **Covariate** | **Definition and data sources** |
| --- | --- |
| Child’s sex | Female, male Birth questionnaire (ABIS) and MBRN (MoBa) |
| Birth year | 1997–1999 (ABIS), 1999–2009 (MoBa) Birth questionnaire (ABIS) and MBRN (MoBa) |
| Parental IBD | Having at least one parent with IBD Birth questionnaire (ABIS) and Norwegian NPR (MoBa) |
| Parental origin of birth | Parental country of birth (ABIS) and parental mother tongue (MoBa) Birth (ABIS) and 15-week pregnancy questionnaire (MoBa) |
| Maternal education level | 9–11, 12, and ≥13 years Birth (ABIS) and 15-week pregnancy questionnaire (MoBa) |
| Maternal comorbidities | Type 1 diabetes, thyroid disease, or rheumatoid arthritis Birth (ABIS) and 15-week pregnancy questionnaire (MoBa) |
| Maternal age at delivery | <17, 18–19, 20–24, 25–29, 30–34, 35–39, 40–44 (excluding <15 and >44) Medical birth registries in ABIS and MoBa |

Type 1 diabetes includes insulin-treated diabetes during pregnancy/at birth, as well as gestational diabetes. Education level ≥13 years includes post-secondary education. ABIS, All Babies in Southeast Sweden; IBD, inflammatory bowel disease; MoBa, the Norwegian Mother, Father and Child Cohort Study; MBRN, medical birth registry of Norway; NPR, national patient register.

**Supplementary Table 6.** Participant baseline characteristics from the ABIS and MoBa cohorts, by tertiles of the Modifiable Health-related Lifestyle Score (MHLS) by 1 year, considering only those with complete lifestyle exposure data

|  |  | **ABIS** |  |  | **MoBa** |  |
| --- | --- | --- | --- | --- | --- | --- |
|  | **Low** | **Medium** | **High** | **Low** | **Medium** | **High** |
| Total n | 3,075 | 1,469 | 2,180 | 14,775 | 21,665 | 22,021 |
| IBD events, n (%) | 43 (1.4) | 16 (1.1) | 20 (0.9) | 108 (0.7) | 129 (0.6) | 100 (0.5) |
| Median follow-up in years (IQR) | 24.4 (23.8–24.8) | 24.3 (23.7–24.8) | 24.3 (23.7–24.8) | 18.5 (16.9–20.0) | 18.1 (16.6–19.7) | 17.8 (16.4–19.3) |
| Median calendar year of birth (range) | 1998 (1997–1999) | 1998 (1997–1999) | 1998 (1997–1999) | 2005 (2002–2009) | 2005 (2002–2009) | 2006 (2002–2009) |
| Sex (%) |  |  |  |  |  |  |
| Female | 1,451 (47.2) | 713 (48.5) | 1,117 (51.2) | 6,915 (46.8) | 10,519 (48.6) | 11,215 (50.9) |
| Male | 1,624 (52.8) | 756 (51.5) | 1,063 (48.8) | 7,860 (53.2) | 11,146 (51.4) | 10,806 (49.1) |
| Parental IBD (%) |  |  |  |  |  |  |
| Yes | 41 (1.3) | 25 (1.8) | 26 (1.2) | 387 (2.6) | 505 (2.3) | 540 (2.5) |
| No | 3,034 (98.7) | 1,443 (98.2) | 2,154 (98.8) | 14,388 (97.4) | 21,160 (97.7) | 21,481 (97.5) |
| Parental country of birth (%) |  |  |  |  |  |  |
| Swedish/Norwegian | 2767 (90.0) | 1,365 (92.9) | 1,979 (90.8) | 13,249 (89.7) | 19,274 (89.0) | 19,466 (88.4) |
| Other | 305 (9.9) | 102 (6.9) | 199 (9.1) | 1,299 (8.8) | 2,021 (9.3) | 2,197 (10.0) |
| Missing | 3 (0.1) | 2 (0.1) | 2 (0.1) | 227 (1.5) | 370 (1.7) | 358 (1.6) |
| Maternal education level (%) |  |  |  |  |  |  |
| ≤11 years | 288 (9.4) | 52 (3.5) | 115 (5.3) | 1,555 (10.5) | 1,029 (4.7) | 574 (2.6) |
| 12 years | 1,824 (59.3) | 819 (55.8) | 1,140 (52.3) | 5,511 (37.3) | 5,865 (27.1) | 4,281 (19.4) |
| ≥13 years | 949 (30.9) | 594 (40.4) | 921 (42.2) | 7,649 (51.8) | 14,704 (67.9) | 17,082 (77.6) |
| Missing | 14 (0.5) | 4 (0.3) | 4 (0.2) | 60 (0.4) | 67 (0.3) | 84 (0.4) |
| Maternal comorbidities (%) |  |  |  |  |  |  |
| Yes | 119 (3.9) | 44 (3.0) | 69 (3.2) | 702 (4.8) | 844 (3.9) | 816 (3.7) |
| No | 2,956 (96.1) | 1,425 (97.0) | 2,111 (96.8) | 14,073 (95.2) | 20,821 (96.1) | 21,205 (96.3) |
| Maternal age (%) |  |  |  |  |  |  |
| <25 years | 540 (17.6) | 212 (14.5) | 238 (10.9) | 1,930 (13.1) | 2,106 (10.3) | 1,304 (6.0) |
| 25–34 years | 2,161 (70.2) | 1,059 (72.1) | 1,636 (75.0) | 10,295 (69.7) | 15,943 (73.6) | 16,608 (75.4) |
| >35 years | 321 (10.4) | 152 (11.7) | 256 (11.7) | 2,544 (17.3) | 3,693 (17.0) | 4,099 (16.8) |
| Missing | 53 (1.7) | 26 (1.8) | 51 (2.3) | 6 (0) | 13 (0.1) | 10 (0) |

Descriptive data are presented as numbers (percentages) unless otherwise stated. Definitions of covariates and exposures are provided in Supplementary Table 5. Maternal comorbidities include type 1 diabetes, thyroid disease, and rheumatoid arthritis. ABIS, All Babies in Southeast Sweden; IBD, inflammatory bowel disease; MoBa, The Norwegian, Mother, Father and Child Cohort Study.

**Supplementary Table 7.** Distribution of modifiable lifestyle factors included in the Modifiable Health-related Lifestyle Score (MHLS) by age 1 year in complete case analyses

| **Cohort** |  | **ABIS** |  |  | **MoBa** |  |
| --- | --- | --- | --- | --- | --- | --- |
|  | **Low  (0–4 points)** | **Medium (4.5 points)** | **High (5–6 points)** | **Low (0–3.5 points)** | **Medium (4–4.5 points)** | **High (5–6 points)** |
| Maternal smoking during pregnancy (%) |  |  |  |  |  |  |
| 0 cigarettes per day | 2,572 (83.6) | 1,419 (96.6) | 2,164 (99.3) | 11,114 (75.2) | 21,185 (97.8) | 21,961 (99.7) |
| 1-5 cigarettes per day | 189 (6.1) | 38 (2.6) | 11 (0.5) | 2,297 (15.5) | 455 (2.1) | 58 (0.3) |
| ≥6 cigarettes per day | 314 (10.2) | 12 (0.8) | 5 (0.2) | 1,364 (9.2) | 25 (0.1) | 2 (0) |
| Delivery mode (%) |  |  |  |  |  |  |
| Vaginal | 2,381 (77.4) | 1,394 (94.9) | 2,149 (98.6) | 9468 (64.1) | 19,035 (87.9) | 21,771 (98.9) |
| Caesarean section | 694 (22.6) | 75 (5.1) | 31 (1.4) | 5,307 (35.9) | 2,630 (12.1) | 250 (1.1) |
| Breastfeeding duration (%) |  |  |  |  |  |  |
| <4 months | 1,707 (55.5) | 360 (24.5) | 312 (14.3) | 9,441 (63.9) | 10,101 (46.6) | 3,883 (17.6) |
| 4-5 months | 1,028 (33.4) | 955 (65.0) | 1,015 (46.6) | 4,961 (33.6) | 9,965 (46.0) | 11,860 (53.9) |
| >6 months | 340 (11.1) | 154 (10.5) | 853 (39.1) | 373 (2.5) | 1,599 (7.4) | 6,278 (28.5) |
| Passive smoking at age 1 year (%) |  |  |  |  |  |  |
| Yes | 330 (10.7) | 18 (1.2) | 5 (0.2) | 10,018 (67.8) | 4,403 (20.3) | 311 (1.4) |
| Diet quality at age 1 year |  |  |  |  |  |  |
| Low | 1,794 (58.3) | 652 (44.4) | 279 (12.8) | 7,704 (52.1) | 8,571 (39.6) | 1,334 (6.1) |
| Medium | 862 (28.0) | 492 (33.5) | 756 (34.7) | 5,522 (37.4) | 8,207 (37.9) | 8,471 (38.5) |
| High | 419 (13.6) | 325 (22.1) | 1,144 (52.5) | 1,549 (10.5) | 4,887 (22.6) | 12,216 (55.5) |
| Missing | 0 (0) | 0 (0) | 1 (0) | 0 (0) | 0 (0) | 0 (0) |
| Antibiotic use at age 1 year (%) |  |  |  |  |  |  |
| Yes | 1,957 (63.6) | 344 (23.4) | 116 (5.3) | 4079 (27.6) | 2,251 (10.4) | 235 (1.1) |
| Sedentary behaviour at age 3 years (%) |  |  |  |  |  |  |
| Low | 901 (29.3) | 487 (33.2) | 714 (32.8) | 5,387 (36.5) | 9,358 (43.2) | 11,185 (50.8) |
| High | 1,076 (35.0) | 509 (34.6) | 741 (34.0) | 5,257 (35.6) | 6,989 (32.3) | 5,771 (26.2) |
| Missing | 1,098 (35.7) | 473 (32.2) | 725 (33.3) | 4,131 (28.0) | 5,318 (24.5) | 5,065 (23.0) |
| Physical activity at age 3 years (%) |  |  |  |  |  |  |
| Low | 1,003 (32.6) | 508 (34.6) | 735 (33.7) | 7,140 (48.3) | 10,887 (50.3) | 10,888 (49.4) |
| High | 1,044 (34.0) | 517 (35.2) | 765 (35.1) | 3,462 (23.4) | 5,420 (25.0) | 6,030 (27.4) |
| Missing | 1,028 (33.4) | 444 (30.2) | 680 (31.2) | 4,173 (28.2) | 5,358 (24.7) | 5,103 (23.2) |
| Body mass index at age 3 years (%) |  |  |  |  |  |  |
| Underweight | 171 (5.6) | 100 (6.8) | 124 (5.7) | 6,016 (40.7) | 9,571 (44.2) | 10,221 (46.4) |
| Normal weight | 1,457 (47.4) | 722 (49.1) | 1,101 (50.5) | 245 (1.7) | 309 (1.4) | 261 (1.2) |
| Overweight/obesity | 346 (11.2) | 159 (10.8) | 225 (10.3) | 2,437 (16.4) | 3,753 (17.5) | 3,643 (16,5) |
| Missing | 1,101 (35.8) | 488 (33.2) | 730 (33.5) | 6,077 (41.1) | 8,032 (37.1) | 7,896 (35.9) |

The tertiles for low, medium, and high Modifiable Health-related Lifestyle Scores by 3 years were 0-4, 4.5-5, and 5.5-7 in ABIS and MoBa and were used to classify participants into three equal groups. ABIS, All Babies in Southeast Sweden; MoBa, the Norwegian Mother, Father and Child Cohort Study.

## **Supplementary Table 8.** Population attributable risk for Modifiable Health-related Lifestyle Score (MHLS) and Optimal Risk Score (ORS) by 1 and 3 years

|  | **Population attributable risk (95% CI)** | | |
| --- | --- | --- | --- |
|  | **IBD (n = 732)** | **CD (n = 293)** | **UC (n = 283)** |
| 1 year |  |  |  |
| MHLS |  |  |  |
| Low-medium to high | 20% (-7–41 %) | 32 % (10–50%) | 4% (-21–25%) |
| ORS |  |  |  |
| Low-medium to high | 14% (1–36%) | 38% (0–62%) | 10% (-18–32%) |
| 3 years |  |  |  |
| MHLS |  |  |  |
| Low-medium to high | 9% (-5–22%) | 18% (-1–35%) | 4% (-17–21%) |
| ORS |  |  |  |
| Low-medium to high | 8% (4–20%) | 14% (-9–33%) | 5% (-34–37%) |

Assuming causal relationships, the population-attributable risk represents the percentage of cases that could have been prevented if individuals in the group with low or medium adherence had adopted a high adherence to health-related modifiable lifestyle factors. Estimates are adjusted for the child’s sex, birth year, parental IBD and country of origin, maternal education level, immune-mediated comorbidities, and age at delivery.

The total number of participants is presented in Figure 2. ABIS, All Babies in Southeast Sweden; CD, Crohn’s disease; CI, confidence interval; IBD, inflammatory bowel disease; MHLS, Modifiable Health-related Lifestyle Score; MoBa, The Norwegian Mother, Father and Child Cohort; ORS, Optimal Risk Score; UC, ulcerative colitis.

## **Supplementary Table 9.** Cohort-specific and pooled analyses of the Modifiable Health-related Lifestyle Score (MHLS) by 1 and 3 years of age in complete-case analyses.

|  |  |  | **ABIS** | |  |  | **MoBa** | | **Pooled** | |
| --- | --- | --- | --- | --- | --- | --- | --- | --- | --- | --- |
| **Exposure** | **N** | **events, n** | **Crude HR  (95% CI)** | **Adjusted HR  (95% CI)** | **N** | **events, n** | **Crude HR  (95% CI)** | **Adjusted HR  (95% CI)** | **Crude HR  (95% CI)** | **Adjusted HR  (95% CI)** |
| **IBD** |  |  |  |  |  |  |  |  |  |  |
| MHLS 1 year |  |  |  |  |  |  |  |  |  |  |
| Low | 3,075 | 43 | Reference | Reference | 8,439 | 70 | Reference | Reference | Reference | Reference |
| Medium | 1,469 | 16 | 0.77 (0.44–1.38) | 0.79 (0.44–1.44) | 13,260 | 80 | 0.84 (0.65–1.09) | 0.84 (0.65–1.10) | 0.83 (0.66–1.05) | 0.84 (0.66–1.06) |
| High | 2,180 | 20 | 0.66 (0.39–1.11) | 0.71 (0.41–1.24) | 13,755 | 69 | 0.67 (0.51–0.88) | 0.67 (0.51–0.89) | 0.67 (0.52–0.85) | 0.68 (0.53–0.87) |
| Per 1-point increase in score |  |  | 0.79 (0.63–0.99) | 0.81 (0.61–1.07) |  |  | 0.86 (0.78–0.96) | 0.86 (0.78–0.96) | 0.85 (0.77–0.93) | 0.85 (0.77–0.95) |
| MHLS 3 years |  |  |  |  |  |  |  |  |  |  |
| Low | 2,259 | 23 | Reference | Reference | 11,826 | 84 | Reference | Reference | Reference | Reference |
| Medium | 2,122 | 22 | 1.01 (0.57–1.24) | 1.04 (0.57–1.89) | 11,136 | 65 | 0.85 (0.11–1.17) | 0.85 (0.61–1.18) | 0.88 (0.67–1.18) | 0.89 (0.67–1.19) |
| High | 1,572 | 15 | 0.94 (0.49–1.10) | 0.98 (0.50–1.94) | 12,490 | 68 | 0.79 (0.57–1.09) | 0.79 (0.57–1.09) | 0.82 (0.61–1.09) | 0.82 (0.61–1.10) |
| Per 1-point increase in score |  |  | 0.90 (0.73–1.02) | 0.94 (0.73–1.19) |  |  | 0.92 (0.82–1.03) | 0.92 (0.82–1.03) | 0.92 (0.83–1.02) | 0.92 (0.83–1.02) |
| **CD** |  |  |  |  |  |  |  |  |  |  |
| MHLS 1 year |  |  |  |  |  |  |  |  |  |  |
| Low | 3,075 | 21 | Reference | Reference | 8,439 | 31 | Reference | Reference | Reference | Reference |
| Medium | 1,469 | 4 | 0.40 (0.14–1.16) | 0.46 (0.15–1.36) | 13,260 | 34 | 0.80 (0.55–1.17) | 0.79 (0.53–1.16) | 0.68 (0.38–1.21) | 0.74 (0.51–1.07) |
| High | 2,180 | 7 | 0.47 (0.20–1.10) | 0.53 (0.23–1.20) | 13,755 | 24 | 0.47 (0.30–0.73) | 0.45 (0.29–0.71) | 0.47 (0.32–0.70) | 0.47 (0.31–0.70) |
| Per 1-point increase in score |  |  | 0.66 (0.47–0.94) | 0.69 (0.46–1.03) |  |  | 0.77 (0.66–0.91) | 0.75 (0.64–0.89) | 0.75 (0.65–0.87) | 0.74 (0.64–0.86) |
| MHLS 3 years |  |  |  |  |  |  |  |  |  |  |
| Low | 2,259 | 8 | Reference | Reference | 11,826 | 40 | Reference | Reference | Reference | Reference |
| Medium | 2,122 | 7 | 0.93 (0.34–2.27) | 0.99 (0.37–2.29) | 11,136 | 22 | 0.60 (0.36–1.43) | 0.59 (0.35–0.98) | 0.66 (0.41–1.04) | 0.66 (0.41–1.04) |
| High | 1,572 | 5 | 0.90 (0.29–2.74) | 0.99 (0.31–3.13) | 12,490 | 27 | 0.66 (0.40–1.25) | 0.62 (0.38–1.02) | 0.69 (0.44–1.09) | 0.67 (0.42–1.05) |
| Per 1-point increase in score |  |  | 0.95 (0.64–1.43) | 0.99 (0.66–1.48) |  |  | 0.87 (0.73–1.07) | 0.95 (0.71–1.01) | 0.88 (0.75–1.03) | 0.87 (0.74–1.02) |
| **UC** |  |  |  |  |  |  |  |  |  |  |
| MHLS 1 year |  |  |  |  |  |  |  |  |  |  |
| Low | 3,075 | 20 | Reference | Reference | 8,439 | 22 | Reference | Reference | Reference | Reference |
| Medium | 1,469 | 9 | 0.94 (0.61–1.68) | 0.90 (0.39–2.06) | 13,260 | 28 | 0.94 (0.61–1.47) | 0.97 (0.62–1.52) | 0.95 (0.64–1.39) | 0.96 (0.64–1.42) |
| High | 2,180 | 13 | 0.92 (0.41–1.54) | 0.98 (0.45–2.16) | 13,755 | 25 | 0.75 (0.47–1.21) | 0.83 (0.51–1.34) | 0.80 (0.54–1.19) | 0.87 (0.58–1.31) |
| Per 1-point increase in score |  |  | 0.93 (0.72–1.14) | 0.97 (0.64–1.47) |  |  | 0.92 (0.77–1.11) | 0.96 (0.79–1.06) | 0.93 (0.79–1.09) | 0.97 (0.64–1.46) |
| MHLS 3 years |  |  |  |  |  |  |  |  |  |  |
| Low | 2,259 | 13 | Reference | Reference | 11,826 | 24 | Reference | Reference | Reference | Reference |
| Medium | 2,122 | 12 | 0.98 (0.45–2.15) | 0.96 (0.42–2.18) | 11,136 | 24 | 1.12 (0.63–1.97) | 1.14 (0.63–2.04) | 1.07 (0.67–1.69) | 1.07 (0.67–1.73) |
| High | 1,572 | 9 | 0.99 (0.43–2.33) | 1.00 (0.41–2.44) | 12,490 | 26 | 1.07 (0.62–1.88) | 1.15 (0.65–2.05) | 1.05 (0.66–1.67) | 1.11 (0.68–1.79) |
| Per 1-point increase in score |  |  | 0.88 (0.66–1.17) | 0.90 (0.63–1.26) |  |  | 0.99 (0.81–1.21) | 1.01 (0.82–1.25) | 0.95 (0.81–1.12) | 0.98 (0.82–1.17) |

Pooled adjusted HR adjusted for the child’s sex, birth year, parental IBD, country of origin, maternal education level, comorbidities, and age at delivery. “Low,” “medium,” and “high” correspond to tertiles of adherence to health-related factors of MHLS (Supplementary Table 3), where “high” represents the group with the healthiest lifestyle. The MHLS ranged from 0 to 6, with higher values indicating greater adherence to health-related factors. ABIS, All Babies in Southeast Sweden; CD, Crohn’s disease; CI, confidence interval; HR, hazard ratio; IBD, inflammatory bowel disease; MHLS, Modifiable Health-related Lifestyle Score; MoBa, the Norwegian Mother, Father and Child Cohort Study; UC, ulcerative colitis.

## **Supplementary Table 10.** Cohort-specific analyses of the Optimal Risk Score (ORS) by 1 and 3 years of age using multiple imputation.

|  |  |  | **ABIS** | |  |  | **MoBa** | |
| --- | --- | --- | --- | --- | --- | --- | --- | --- |
| **Exposure** | **N** | **events, n** | **Crude HR  (95% CI)** | **Adjusted HR  (95% CI)** | **N** | **events, n** | **Crude HR  (95% CI)** | **Adjusted HR  (95% CI)** |
| **IBD** |  |  |  |  |  |  |  |  |
| **ORS 1 year** |  |  |  |  |  |  |  |  |
| Low | 3,976 | 47 | Reference | Reference | 35,798 | 235 | Reference | Reference |
| Medium | 5,151 | 49 | 0.79 (0.47–1.32) | 0.80 (0.48-1.35) | 41,349 | 228 | 0.86 (0.71-1.04) | 0.86 (0.71-1.05) |
| High | 7,091 | 57 | 0.67 (0.42–1.07) | 0.69 (0.43-1.10) | 24,122 | 118 | 0.80 (0.63-1.02) | 0.80 (0.63-1.02) |
| Per 10-points decrease in score |  |  | 0.89 (0.81–0.97) | 0.89 (0.82-0.98) |  |  | 0.94 (0.90-0.98) | 0.94 (0.90-0.98) |
| ORS 3 years |  |  |  |  |  |  |  |  |
| Low | 4,622 | 54 | Reference | Reference | 30,361 | 207 | Reference | Reference |
| Medium | 4,976 | 48 | 0.83 (0.52-1.23) | 0.84 (0.52-1.34) | 32,499 | 170 | 0.81 (0.65-1.02) | 0.80 (0.64-1.02) |
| High | 6,615 | 49 | 0.63 (0.39-1.00) | 0.64 (0.39-1.03) | 27,978 | 144 | 0.82 (0.65-1.02) | 0.81 (0.64-1.02) |
| Per 10-points decrease in score |  |  | 0.90 (0.82-0.98) | 0.90 (0.82-0.98) |  |  | 0.97 (0.93-1.00) | 0.96 (0.93-1.00) |
| CD |  |  |  |  |  |  |  |  |
| ORS 1 year |  |  |  |  |  |  |  |  |
| Low | 6,204 | 25 | Reference | Reference | 35,799 | 102 | Reference | Reference |
| Medium | 7,193 | 28 | 0.96 (0.50-1.83) | 0.98 (0.51-1.87) | 41,351 | 94 | 0.82 (0.60-1.10) | 0.78 (0.58-1.06) |
| High | 2,821 | 4 | 0.32 (0.10-1.02) | 0.33 (0.10-1.02) | 24,120 | 41 | 0.63 (0.43-0.93) | 0.59 (0.39-0.87) |
| Per 10-points decrease in score |  |  | 0.89 (0.77-1.03) | 0.90 (0.77-1.04) |  |  | 0.90 (0.85-0.95) | 0.89 (0.84-0.94) |
| ORS 3 years |  |  |  |  |  |  |  |  |
| Low | 5,475 | 22 | Reference | Reference | 30,361 | 91 | Reference | Reference |
| Medium | 5,887 | 18 | 0.73 (0.32-1.62) | 0.73 (0.32-1.64) | 32,498 | 71 | 0.76 (0.54-1.07) | 0.72 (0.51-1.01) |
| High | 4,852 | 16 | 0.80 (0.37-1.71) | 0.79 (0.36-1.72) | 27,979 | 56 | 0.71 (0.49-1.01) | 0.65 (0.45-0.94) |
| Per 10-points decrease in score |  |  | 0.90 (0.78-1.04) | 0.89 (0.78-1.04) |  |  | 0.95 (0.90-1.00) | 0.93 (0.89-0.99) |
| UC |  |  |  |  |  |  |  |  |
| ORS 1 year |  |  |  |  |  |  |  |  |
| Low | 6,205 | 39 | Reference | Reference | 35,800 | 81,55 | Reference | Reference |
| Medium | 7,192 | 33 | 0.74 (0.44-1.23) | 0.76 (0.45-1.27) | 41,351 | 77 | 0.84 (0.60-1.18) | 0.85 (0.1-1.20) |
| High | 2,821 | 14 | 0.76 (0.40-1.45) | 0.80 (0.42-1.53) | 24,118 | 39 | 0.78 (0.52-1.19) | 0.80 (0.52-1.22) |
| Per 10-points decrease in score |  |  | 0.90 (0.80-1.01) | 0.91 (0.80-1.02) |  |  | 0.95 (0.89-1.02) | 0.96 (0.89-1.02) |
| ORS 3 years |  |  |  |  |  |  |  |  |
| Low | 5,475 | 31 | Reference | Reference | 30,361 | 66 | Reference | Reference |
| Medium | 5,885 | 36 | 1.07 (0.62-1.83) | 1.08 (0.63-1.87) | 32,499 | 57 | 0.88 (0.60-1.29) | 0.90 (0.61-1.33) |
| High | 4,852 | 19 | 0.67 (0.36-1.28) | 0.70 (0.37-1.34) | 27,978 | 55 | 1.01 (0.68-1.51) | 1.04 (0.69-1.57) |
| Per 10-points decrease in score |  |  | 0.95 (0.84-1.07) | 0.96 (0.85-1.08) |  |  | 0.99 (0.93-1.05) | 0.99 (0.93-1.06) |

The ORS ranged from 0 to 100, with higher values indicating a higher predicted risk of IBD; thus, lower ORS values correspond to a healthier lifestyle. The number of participants (N) and events (n) represent the mean values across the imputed datasets. Analyses were adjusted for the child’s sex, birth year, parental IBD, country of origin, maternal education level, maternal comorbidities, and maternal age at delivery (Supplementary Table 3). CI, confidence interval; HR, hazard ratio.

## **Supplemental Table 11.** Pooled adjusted hazard ratios in analyses with and without delivery mode for the Modifiable Health-related Lifestyle Score (MHLS) by 1 year in complete case data

|  | **N** | **Events, n** | **With delivery mode**  **aHR (95%CI)^a^** | **Without a delivery mode aHR (95%CI)^b^** |
| --- | --- | --- | --- | --- |
| Inflammatory bowel disease |  |  |  |  |
| Low | 11,514 | 113 | Reference | Reference |
| Medium | 14,729 | 96 | 0.85 (0.71–1.02) | 0.84 (0.65–1.08) |
| High | 15,935 | 89 | 0.76 (0.59–0.98) | 0.76 (0.61–0.95) |
| Crohn’s disease |  |  |  |  |
| Low | 11,514 | 52 | Reference | Reference |
| Medium | 14,729 | 38 | 0.71 (0.53–0.93) | 0.69 (0.39–1.21) |
| High | 15,935 | 31 | 0.61 (0.41–0.91) | 0.55 (0.38–0.78) |
| Ulcerative colitis |  |  |  |  |
| Low | 11,514 | 42 | Reference | Reference |
| Medium | 14,729 | 37 | 0.93 (0.70–1.24) | 1.01 (0.66–1.56) |
| High | 15,935 | 38 | 0.92 (0.63–1.34) | 1.06 (0.73–1.55) |

CD, Crohn’s disease; aHR: adjusted hazard ratio; CI, confidence interval; IBD, inflammatory bowel disease; UC, ulcerative colitis. For a description of covariates, see Supplementary Table 3.

^a^Modifiable Health-related Lifestyle Score aged 1 year, including delivery mode, pooled HR adjusted for the child’s sex, birth year, parental IBD, country of origin, maternal education level, comorbidities, and age at delivery (Figure 1).

^b^Modifiable Health-related Lifestyle Score aged 1 year excluding delivery mode, pooled HR adjusted for the child’s sex, birth year, parental IBD, country of origin, maternal education level, comorbidities, and age at delivery.

## **Supplementary Table 12.** Pooled adjusted hazard ratios with and without additional adjustment for full breastfeeding duration for the Modifiable Health-related Lifestyle Score (MHLS) at 3 years of age, and risk of IBD, CD, and UC in imputed datasets.

|  | **N** | **Events, n** | **Without adjustment for breastfeeding**  **aHR (95%CI)^a^** | **N** | **Events, n** | **With adjustment for breastfeeding aHR (95%CI)^b^** |
| --- | --- | --- | --- | --- | --- | --- |
| Inflammatory bowel disease |  |  |  |  |  |  |
| Low | 45440 | 303 | Reference | 45437 | 301 | Reference |
| Medium | 38872 | 238 | 0.98 (0.79–1.21) | 38877 | 238 | 0.99 (0.80–1.23) |
| High | 32969 | 184 | 0.85 (0.63–1.15) | 32967 | 186 | 0.92 (0.74–1.14) |
| Crohn’s disease |  |  |  |  |  |  |
| Low | 45459 | 236 | Reference | 45483 | 124 | Reference |
| Medium | 38900 | 140 | 0.95 (0.67–1.35) | 38881 | 97 | 0.93 (0.68–1.26) |
| High | 32922 | 63 | 0.79 (0.56–1.13) | 32917 | 71 | 0.82 (0.58–1.16) |
| Ulcerative colitis |  |  |  |  |  |  |
| Low | 45482 | 116 | Reference | 45479 | 117 | Reference |
| Medium | 38897 | 93 | 1.02 (0.71–1.46) | 38902 | 92 | 1.00 (0.70–1.42) |
| High | 32901 | 72 | 1.03 (0.72–1.46) | 32900 | 72 | 1.00 (0.70–1.44) |

^a^Pooled HR adjusted for the child’s sex, birth year, parental IBD, country of origin, maternal education level, comorbidities, and age at delivery (Figure 2).

^b^Pooled HR adjusted for the child’s sex, birth year, parental IBD, country of origin, maternal education level, comorbidities, age at delivery, and full breastfeeding duration. For a description of covariates, see Supplementary Table 3.

MHLS 3 years do not include breastfeeding as a health-related lifestyle factor. The number of participants (N) and events (n) represent the mean values across the imputed datasets.

CD, Crohn’s disease; aHR: adjusted hazard ratio; CI, confidence interval; IBD, inflammatory bowel disease; UC, ulcerative colitis.

## **Supplementary Table 13.** Pooled adjusted hazard ratios restricted to childhood-onset IBD and excluding those with VEO-IBD for the Modifiable Health-related Lifestyle Score (MHLS) at 1 and 3 years of age, and risk of IBD in imputed datasets.

|  | **N** | **Events, n** | **Main findings**  **aHR (95%CI)^a^** | **N** | **Events, n** | **Restricting to childhood-onset IBD aHR (95%CI)^b^** | **N** | **Events, n** | **Excluding those with VEO-IBD aHR (95%CI)^c^** |
| --- | --- | --- | --- | --- | --- | --- | --- | --- | --- |
| MHLS 1 year |  |  |  |  |  |  |  |  |  |
| Low | 51,704 | 364 | Reference | 51,695 | 257 | Reference | 51,685 | 354 | Reference |
| Medium | 47,768 | 274 | 0.85 (0.71–1.02) | 47,790 | 19 | 0.81 (0.66–1.00) | 47,757 | 263 | 0.83 (0.69–0.99) |
| High | 18,015 | 934 | 0.76 (0.59–0.98) | 17,997 | 62 | 0.60 (0.29–1.27) | 18,022 | 91 | 0.77 (0.60–0.99) |
| MHLS 3 years |  |  |  |  |  |  |  |  |  |
| Low | 45,440 | 303 | Reference | 45,420 | 207 | Reference | 45,481 | 293 | Reference |
| Medium | 38,872 | 238 | 0.97 (0.79–1.19) | 38,891 | 166 | 0.95 (0.73–1.24) | 38,892 | 231 | 0.97 (0.80–1.18) |
| High | 32,969 | 184 | 0.85 (0.63–1.13) | 32,899 | 128 | 0.87 (0.67–1.14) | 32,889 | 183 | 0.92 (0.69–1.22) |

^a^Pooled HR adjusted for the child’s sex, birth year, parental IBD, country of origin, maternal education level, comorbidities, and age at delivery (Figure 2).

^b^Pooled HR adjusted for the child’s sex, birth year, parental IBD, country of origin, maternal education level, comorbidities, and age at delivery, restricted to those with childhood-onset IBD (<18 years). For a description of covariates, see Supplementary Table 3.

^c^Pooled HR adjusted for the child’s sex, birth year, parental IBD, country of origin, maternal education level, comorbidities, and age at delivery, excluding those with VEO-IBD (<6 years). For a description of covariates, see Supplementary Table 3.

The number of participants (N) and events (n) represent the mean values across the imputed datasets.

aHR: adjusted hazard ratio; CI, confidence interval; IBD, inflammatory bowel disease; VEO-IBD, very-early onset inflammatory bowel disease.

**Supplementary Table 14.** Associations between Health-related Lifestyle Score (MHLS) at 1 of age and risk of IBD and CD after excluding antibiotics from the score in imputed datasets.

|  | **N** | **Events, n** | **Main findings**  **aHR (95%CI)^a^** | **MHLS 1 year without antibiotics aHR (95%CI)^b^** |
| --- | --- | --- | --- | --- |
| MHLS 1 year and IBD risk | |  |  |  |
| Low | 51,704 | 364 | Reference | Reference |
| Medium | 47,768 | 274 | 0.85 (0.71–1.02) | 0.85 (0.71–1.03) |
| High | 18,015 | 934 | 0.76 (0.59–0.98) | 0.82 (0.68–0.99) |
| MHLS at 1 year and CD risk | |  |  |  |
| Low | 51,681 | 160 | Reference | Reference |
| Medium | 47,805 | 100 | 0.71 (0.53–0.93) | 0.76 (0.55–1.06) |
| High | 18,002 | 33 | 0.61 (0.41–0.91) | 0.67 (0.49–0.90) |

^a^Pooled HR adjusted for the child’s sex, birth year, parental IBD, country of origin, maternal education level, comorbidities, and age at delivery (Figure 2).

^b^Pooled HR adjusted for the child’s sex, birth year, parental IBD, country of origin, maternal education level, comorbidities, and age at delivery, when antibiotics were excluded from the MHLS 1 year score. For a description of covariates, see Supplementary Table 3.

The number of participants (N) and events (n) represent the mean values across the imputed datasets.

aHR: adjusted hazard ratio; CD, Crohn’s disease; CI, confidence interval; IBD, inflammatory bowel disease.

## **Supplementary Table 15.** Associations between Health-related Lifestyle Score (MHLS) at 1 of age and risk of IBD and CD after additional adjustments for parental household income in imputed datasets.

|  | **N** | **Events, n** | **Main findings**  **aHR (95%CI)^a^** | **N** | **Events, n** | **MHLS 1 year adjusting for parental income aHR (95%CI)^b^** |
| --- | --- | --- | --- | --- | --- | --- |
| MHLS 1 year and IBD risk | |  |  |  |  |  |
| Low | 51,704 | 364 | Reference | 51,697 | 364 | Reference |
| Medium | 47,768 | 274 | 0.85 (0.71–1.02) | 47,769 | 274 | 0.83 (0.71–0.99) |
| High | 18,015 | 934 | 0.76 (0.59–0.98) | 18,020 | 94 | 0.78 (0.60–1.00) |
| MHLS 1 year and CD risk | |  |  |  |  |  |
| Low | 51,681 | 160 | Reference | 51,706 | 158 | Reference |
| Medium | 47,805 | 100 | 0.71 (0.53–0.93) | 47,790 | 1001 | 0.67 (0.51–0.88) |
| High | 18,002 | 33 | 0.61 (0.41–0.91) | 17,991 | 34 | 0.59 (0.40–0.88) |

^a^Pooled HR adjusted for the child’s sex, birth year, parental IBD, country of origin, maternal education level, comorbidities, and age at delivery (Figure 2).

^b^Pooled HR adjusted for the child’s sex, birth year, parental IBD, country of origin, maternal education level, comorbidities, and age at delivery, when antibiotics were excluded from the MHLS 1 year score. For a description of covariates, see Supplementary Table 3.

The number of participants (N) and events (n) represent the mean values across the imputed datasets.

aHR: adjusted hazard ratio; CD, Crohn’s disease; CI, confidence interval; IBD, inflammatory bowel disease; UC, ulcerative colitis.

## **Supplementary Table 16.** E-values for associations between MHLS and ORS at 1 year and risk of IBD and CD, based on pooled analyses across the ABIS and MoBa cohorts.

| Exposure | **IBD E-value (95% CI)** | **CD E-value (95% CI)** |
| --- | --- | --- |
| MHLS, High vs low | 1.92 (1.16, 2.66) | 2.90 (1.60, 4.70) |
| ORS, High vs low | 1.92 (1.28, 2.55) | 2.30 (1.32, 3.49) |

E-values represent the minimum strength of association that an unmeasured confounder would need to have with both the exposure and the outcome to fully explain the observed associations, conditional on measured covariates.

Estimates are adjusted for the child’s sex, birth year, parental IBD, parental country of origin, maternal education level, immune-mediated comorbidities, and maternal age at delivery.

CD, Crohn’s disease; CI, confidence interval; IBD, inflammatory bowel disease; MHLS, Modifiable Health-related Lifestyle Score; ORS, Optimal Risk Score.

## **Supplementary Table 17.** The RECORD statement checklist

|  | **Item No.** | **STROBE items** | **Location in manuscript where items are reported** | **RECORD items** | **Location in manuscript where items are reported** |
| --- | --- | --- | --- | --- | --- |
| **Title and abstract** | | | | | |
|  | 1 | (a) Indicate the study’s design with a commonly used term in the title or the abstract (b) Provide in the abstract an informative and balanced summary of what was done and what was found |  | RECORD 1.1: The type of data used should be specified in the title or abstract. When possible, the name of the databases used should be included.  RECORD 1.2: If applicable, the geographic region and timeframe within which the study took place should be reported in the title or abstract.  RECORD 1.3: If linkage between databases was conducted for the study, this should be clearly stated in the title or abstract. | Page 1 and 3.  RECORD 1.1: Due to word limitations, we were not able to include type of data in the title. However, its stated in the abstract on page 3.  Record 1.2: Page 3  RECORD: 1.3: Page 3 |
| **Introduction** | | | | | |
| Background rationale | 2 | Explain the scientific background and rationale for the investigation being reported |  |  | Page 4 |
| Objectives | 3 | State specific objectives, including any prespecified hypotheses |  |  | Page 4 |
| **Methods** | | | | | |
| Study Design | 4 | Present key elements of study design early in the paper |  |  | Page 5 |
| Setting | 5 | Describe the setting, locations, and relevant dates, including periods of recruitment, exposure, follow-up, and data collection |  |  | Page 5 |
| Participants | 6 | *(a) Cohort study* - Give the eligibility criteria, and the sources and methods of selection of participants. Describe methods of follow-up  *Case-control study* - Give the eligibility criteria, and the sources and methods of case ascertainment and control selection. Give the rationale for the choice of cases and controls  *Cross-sectional study* - Give the eligibility criteria, and the sources and methods of selection of participants  *(b) Cohort study* - For matched studies, give matching criteria and number of exposed and unexposed  *Case-control study* - For matched studies, give matching criteria and the number of controls per case |  | RECORD 6.1: The methods of study population selection (such as codes or algorithms used to identify subjects) should be listed in detail. If this is not possible, an explanation should be provided.  RECORD 6.2: Any validation studies of the codes or algorithms used to select the population should be referenced. If validation was conducted for this study and not published elsewhere, detailed methods and results should be provided.  RECORD 6.3: If the study involved linkage of databases, consider use of a flow diagram or other graphical display to demonstrate the data linkage process, including the number of individuals with linked data at each stage. | Page 5  RECORD 6.1: Page 5  RECORD 6.2: Page 5-6  RECORD 6.3: Described in text on page 5 |
| Variables | 7 | Clearly define all outcomes, exposures, predictors, potential confounders, and effect modifiers. Give diagnostic criteria, if applicable. |  | RECORD 7.1: A complete list of codes and algorithms used to classify exposures, outcomes, confounders, and effect modifiers should be provided. If these cannot be reported, an explanation should be provided. | Page 5 and Supplementary Table 2 |
| Data sources/ measurement | 8 | For each variable of interest, give sources of data and details of methods of assessment (measurement).  Describe comparability of assessment methods if there is more than one group |  |  | Page 6 and Supplementary Table 3 |
| Bias | 9 | Describe any efforts to address potential sources of bias |  |  | Page 17-19 |
| Study size | 10 | Explain how the study size was arrived at |  |  | Page 11 and Figure 1 |
| Quantitative variables | 11 | Explain how quantitative variables were handled in the analyses. If applicable, describe which groupings were chosen, and why |  |  | Page 7-10 |
| Statistical methods | 12 | (a) Describe all statistical methods, including those used to control for confounding  (b) Describe any methods used to examine subgroups and interactions  (c) Explain how missing data were addressed  (d) *Cohort study* - If applicable, explain how loss to follow-up was addressed  *Case-control study* - If applicable, explain how matching of cases and controls was addressed  *Cross-sectional study* - If applicable, describe analytical methods taking account of sampling strategy  (e) Describe any sensitivity analyses |  |  | Page 9-10 |
| Data access and cleaning methods |  | .. |  | RECORD 12.1: Authors should describe the extent to which the investigators had access to the database population used to create the study population.  RECORD 12.2: Authors should provide information on the data cleaning methods used in the study. | RECORD 12.1: Page 5  RECORD 12.2: No data cleaning was made due to 100% linkage success rate |
| Linkage |  | .. |  | RECORD 12.3: State whether the study included person-level, institutional-level, or other data linkage across two or more databases. The methods of linkage and methods of linkage quality evaluation should be provided. | Page 5 |
| **Results** | | | | | |
| Participants | 13 | (a) Report the numbers of individuals at each stage of the study (*e.g.*, numbers potentially eligible, examined for eligibility, confirmed eligible, included in the study, completing follow-up, and analysed)  (b) Give reasons for non-participation at each stage.  (c) Consider use of a flow diagram |  | RECORD 13.1: Describe in detail the selection of the persons included in the study (*i.e.,* study population selection) including filtering based on data quality, data availability and linkage. The selection of included persons can be described in the text and/or by means of the study flow diagram. | Page 5 and Figure 1 |
| Descriptive data | 14 | (a) Give characteristics of study participants (*e.g.*, demographic, clinical, social) and information on exposures and potential confounders  (b) Indicate the number of participants with missing data for each variable of interest  (c) *Cohort study* - summarise follow-up time (*e.g.*, average and total amount) |  |  | Page 11, Table 1 |
| Outcome data | 15 | *Cohort study* - Report numbers of outcome events or summary measures over time  *Case-control study* - Report numbers in each exposure category, or summary measures of exposure  *Cross-sectional study* - Report numbers of outcome events or summary measures |  |  | Page 11, Figure 1 |
| Main results | 16 | (a) Give unadjusted estimates and, if applicable, confounder-adjusted estimates and their precision (e.g., 95% confidence interval). Make clear which confounders were adjusted for and why they were included  (b) Report category boundaries when continuous variables were categorized  (c) If relevant, consider translating estimates of relative risk into absolute risk for a meaningful time period |  |  | Page 11-12 |
| Other analyses | 17 | Report other analyses done—e.g., analyses of subgroups and interactions, and sensitivity analyses |  |  | Page 13-14 |
| **Discussion** | | | | | |
| Key results | 18 | Summarise key results with reference to study objectives |  |  | Page 15 |
| Limitations | 19 | Discuss limitations of the study, taking into account sources of potential bias or imprecision. Discuss both direction and magnitude of any potential bias |  | RECORD 19.1: Discuss the implications of using data that were not created or collected to answer the specific research question(s). Include discussion of misclassification bias, unmeasured confounding, missing data, and changing eligibility over time, as they pertain to the study being reported. | Page 17-20 |
| Interpretation | 20 | Give a cautious overall interpretation of results considering objectives, limitations, multiplicity of analyses, results from similar studies, and other relevant evidence |  |  | Page 15-17 |
| Generalisability | 21 | Discuss the generalisability (external validity) of the study results |  |  | Page 18 |
| **Other Information** | | | | | |
| Funding | 22 | Give the source of funding and the role of the funders for the present study and, if applicable, for the original study on which the present article is based |  |  | Page 1-2 |
| Accessibility of protocol, raw data, and programming code |  | .. |  | RECORD 22.1: Authors should provide information on how to access any supplemental information such as the study protocol, raw data, or programming code. | Page 2 |

*Reference: Benchimol EI, Smeeth L, Guttmann A, Harron K, Moher D, Petersen I, Sørensen HT, von Elm E, Langan SM, the RECORD Working Committee. The REporting of studies Conducted using Observational Routinely-collected health Data (RECORD) Statement. *PLoS Medicine* 2015; in press.

*Checklist is protected under Creative Commons Attribution ([CC BY](http://creativecommons.org/licenses/by/4.0/)) license.

**References**

1.	Santos MPC, Gomes C, Torres J. Familial and ethnic risk in inflammatory bowel disease. Ann Gastroenterol 2018;31:14-23.

2.	Barclay K, Myrskylä M. Maternal age and offspring health and health behaviours in late adolescence in Sweden. SSM Popul Health 2016;2:68-76.

3.	Sun J, Yao J, Olén O, et al. Bidirectional association between inflammatory bowel disease and type 1 diabetes: a nationwide matched cohort and case-control study. Lancet Reg Health Eur 2024;46:101056.

4.	Chen Y, Chen L, Xing C, et al. The risk of rheumatoid arthritis among patients with inflammatory bowel disease: a systematic review and meta-analysis. BMC Gastroenterol 2020;20:192.

5.	Sigvardsson I, Størdal K, Östensson M, et al. Childhood Socioeconomic Characteristics and Risk of Inflammatory Bowel Disease: A Scandinavian Birth Cohort Study. Inflamm Bowel Dis 2024;30:1801-1811.

6.	Agrawal M, Corn G, Shrestha S, et al. Inflammatory bowel diseases among first-generation and second-generation immigrants in Denmark: a population-based cohort study. Gut 2021;70:1037-1043.

7.	D'Souza S, Levy E, Mack D, et al. Dietary patterns and risk for Crohn's disease in children. Inflamm Bowel Dis 2008;14:367-73.

8.	Ho FF, Gao YY, Chen Y, et al. Association of Healthy Lifestyle Behaviours With Incident Inflammatory Bowel Disease: A Population-Based Prospective Cohort Study. Aliment Pharmacol Ther 2025;61:1519-1531.

9.	Sun Y, Yuan S, Chen X, et al. The Contribution of Genetic Risk and Lifestyle Factors in the Development of Adult-Onset Inflammatory Bowel Disease: A Prospective Cohort Study. Am J Gastroenterol 2023;118:511-522.

10.	Lopes EW, Chan SSM, Song M, et al. Lifestyle factors for the prevention of inflammatory bowel disease. Gut 2022.

11.	Larsen JH, Andersen S, Perminow G, Stangeland HM, Mårild K, Stabell N, et al. Higher incidence of childhood-onset inflammatory bowel disease by increasing latitude in Norway, but stable incidence by age for cohorts born 2004-2012 [Accepted for Acta Paediatrica]. 2024.

12.	Östensson M, Björkqvist O, Guo A, et al. Epidemiology, validation, and clinical characteristics of inflammatory bowel disease: the ABIS birth cohort study. BMC Gastroenterol 2023;23:199.

13.	Sigvardsson I, Ludvigsson J, Andersson B, et al. Tobacco smoke exposure in early childhood and later risk of inflammatory bowel disease: A Scandinavian birth cohort study. J Crohns Colitis 2024.

14.	Agrawal M, Størdal K, Vinkel Hansen A, et al. Breastfeeding Duration Is Not Associated With Offspring Inflammatory Bowel Disease Risk in Three Population-Based Birth Cohorts. Clin Gastroenterol Hepatol 2024;22:2459-2467.e16.

15.	Guo A, Ludvigsson J, Brantsæter AL, et al. Early-life diet and risk of inflammatory bowel disease: a pooled study in two Scandinavian birth cohorts. Gut 2024;73:590-600.

16.	Mårild K, Lerchova T, Östensson M, et al. Early-Life Infections, Antibiotics and Later Risk of Childhood and Early Adult-Onset Inflammatory Bowel Disease: Pooled Analysis of Two Scandinavian Birth Cohorts. Aliment Pharmacol Ther 2025;61:323-334.

17.	Lerchova T, Östensson M, Sigvardsson I, et al. Physical activity in childhood and later risk of inflammatory bowel disease: A Scandinavian birth cohort study. United European Gastroenterol J 2023;11:874-883.

18.	Lerchova T, Ludvigsson J, Mårild S, et al. Early-Life Body Mass Index and Inflammatory Bowel Disease Risk: A Scandinavian Birth Cohort Study. Inflamm Bowel Dis 2025.

19.	Cole TJ, Lobstein T. Extended international (IOTF) body mass index cut-offs for thinness, overweight and obesity. Pediatr Obes 2012;7:284-94.
